# Genome Sequencing Reveals the Complex Polysaccharide-Degrading Ability of Novel Deep-Sea Bacterium *Flammeovirga pacifica* WPAGA1

**DOI:** 10.3389/fmicb.2017.00600

**Published:** 2017-04-10

**Authors:** Boliang Gao, Min Jin, Li Li, Wu Qu, Runying Zeng

**Affiliations:** ^1^School of Life Sciences, Xiamen UniversityXiamen, China; ^2^State Key Laboratory Breeding Base of Marine Genetic Resource, Third Institute of Oceanography, SOAXiamen, China; ^3^Fujian Collaborative Innovation Center for Exploitation and Utilization of Marine Biological ResourcesXiamen, China; ^4^South China Sea Bio-Resource Exploitation and Utilization Collaborative Innovation CenterGuangzhou, China

**Keywords:** genome sequencing, *Flammeovirga pacifica* WPAGA1, polysaccharide-degrading, metabolic pathway, glycometabolism

## Abstract

*Flammeovirga pacifica* strain WPAGA1 is a Gram-negative, polysaccharide-degrading bacterium isolated from the marine sediment of the West Pacific Ocean. This strain is a cosmopolitan marine bacterium that uses complex polysaccharides as exclusive source of carbon and energy and plays a key role in the marine carbon cycle. Genome sequence analysis of strain WPAGA1 revealed that the assembled fine genome contains 6,610,326 bp with 32.89% G+C content, 5036 open reading frames (ORFs) and abundant genomic elements. Amongst these ORFs, 1022 genes encoding carbohydrate enzymes were found in the *F. pacifica* WPAGA1 genome. In addition, abundant putative enzymes involved in degrading polysaccharide were found. These enzymes include amylase, xylosidase, cellulase, alginate lyase, pectate lyase, rhamnogalacturonan lyase, chitinase, carrageenase, heparinase and fucosidase. To further investigate the use of these polysaccharides in strain WPAGA1, a schematic of various polysaccharide-degrading metabolic pathways were deduced from the genome sequence. This study showed that strain WPAGA1 may serve as a potential candidate for research of glycometabolism and have potential biotechnological and industrial applications and play key roles in the marine carbon cycle.

## Introduction

The marine ecosystem covers more than 70% of the Earth's surface and moderates the Earth's climate. This ecosystem plays important roles in the water, carbon and nitrogen cycles. Seaweed includes red, brown and green algae and occupies a wide range of marine ecological niches and serves as the base of the marine food chain (Šesták et al., 1996). Thus, seaweed biolysis is essential for marine ecology and is a key step in material cycles, especially the carbon cycle. Complex polysaccharides, including agar, alginate, chitin, cellulose, pectin and porphyrin, are the major components of seaweed cell walls and intercellular spaces (Heath, 1971; Rochas et al., 1986; Ray and Lahaye, 1995; Martone et al., 2009) that are highly difficult to degrade. In marine environments, a lot of algal polysaccharides-degrading bacteria have been isolated and revealed as the key players in the algal biomass recycling and in the global carbon cycle (Ensor et al., 1999; de Vries and Visser, 2001; Martin et al., 2015). In deep-sea sediments, there are mass of plants and animals debris deposited from the upper ocean, including seaweed debris which provides the carbon sources for polysaccharide-degrading bacteria. Thus, these complex polysaccharide-degrading bacteria are also abundant in deep sea sediments and also play an important role in the carbon cycle of deep sea. On the other hand, algal oligosaccharides were reported to possess diverse physiological and biological functions and hold potential applications in food, cosmetic and medical industries (Bateman and Basham, 1976; Varki, 1993; Yoshizawa et al., 1995; Crittenden and Playne, 1996; Kobayashi et al., 1997; Jang et al., 2009). Therefore, the demand for algal oligosaccharides is rising. Compared with chemical treatment, biodegradation is an efficient and clean strategy for producing the algal oligosaccharides from algal polysaccharides. Hence, the search of novel microorganisms that efficiently degrade polysaccharides is becoming increasingly crucial.

As a typical genus of polysaccharide-degrading bacteria, *Flammeovirga* is a newly defined member of the family *Flammeovirgaceae* of the class α-Proteobacteria. Five species have been reported in this genus, namely, *F. aprica* (Nakagawa et al., 1997), *F. arenaria, F. yaeyamensis* (Takahashi et al., 2006), *F. kamogawensis* (Hosoya and Yokota, 2007), and *F. pacifica* (Xu et al., 2012). All of these strains can degrade complex polysaccharides. For example, Han et al. isolated a *Flammeovirga* strain, namely *Flammeovirga* sp. MY04, and described the extracellular agarase system. Yang et al. (2011) investigated a gene and the biochemical characteristics of an agarase, AgaYT, from the *F. yaeyamensis* strain YT. This enzyme could degrade agar, a marine-type complex polysaccharide. Similar to most strains of *Flammeovirga*, our group isolated novel species from deep-sea sediment (Xu et al., 2012), specifically, *F. pacifica* WPAGA1, and obtained novel agarase AgaP4383 (Hou et al., 2015), which showed excellent ability to degrade agar. These strains belonging to *Flammeovirga* may play important roles in the marine ecosystem, especially the carbon cycle. However, given the lack of genomic information for these species, further understanding of the polysaccharide-degrading ability of these bacteria, as well as their roles in the marine ecosystem, is difficult.

Herein, we report the genome sequence of the novel deep-sea bacteria *F. pacifica* WPAGA1. Genomic analysis yielded unprecedented insight into the bacterium's capacity for degrading and utilizing complex polysaccharides and deduced the metabolic pathways of various polysaccharides in *F. pacifica* WPAGA1, and the strain WPAGA1 is suggested as a potential model for glycometabolism investigation. The genomic data and functional analysis provided us with an invaluable knowledge base essential in revealing the mechanisms and maintenance of the balance in the marine carbon cycle. Our results also offered theoretical basis for this strain's application in industrial production.

## Materials and methods

### Complex polysaccharides-degrading ability of *F. pacifica* WPAGA1

Agar is a kind of complex polysaccharide that exists widely in the marine environment and is hard to degrade. *Flammeovirga pacifica* WPAGA1, which is isolated from deep-sea sediment of west Pacific Ocean (157° 24′ 31″ E 19° 30′ 30″ N) at the depth of 5,378 m, was cultured as described previously (Xu et al., 2012). To evaluate the ability of the bacterial isolate to utilize agar, *F. pacifica* WPAGA1 was grown on modified 2216E medium that contains 0.2% yeast extract, sea water and 2% agar. Then, we observed agar degradation termly. After 36 h, the medium was processed using 2% (m/v) iodine staining.

For additional analysis, the agarose-utilizing ability of the crude extracellular enzymes of *F. pacifica* WPAGA1 were prepared. The WPAGA1 strain was cultured at 28°C for 48 h in 1,500 mL of 2216E medium, and the following preparation was performed on ice. The cultures were centrifuged at 12,000 × g for 15 min, and ammonium sulfate was added to the supernatant to reach 80% saturation. After 4 h incubation to precipitate proteins, the mixture was centrifuged at 15,000 × g for 30 min. The pellet was then dissolved in 10 mL 10 mM PBS buffer (pH 7.4), and the resultant suspension was dialyzed three times against the same buffer for 6 h. Then, the dialyzed enzyme solution was stored in 20% glycerol (v/v) at −20°C. The extracellular agarase system in the crude enzyme extracts was analyzed to identify the agarase components. Crude enzyme extracts (8 μL each) were each mixed with 4 μL 3 × loading buffer and separated by 12% non-denaturing polyacrylamide gel electrophoresis (PAGE). After electrophoresis, the gel was washed three times with 10 mM PBS buffer (pH 7.4) for total of 30 min. The gel was then overlaid onto a 2% agar sheet and incubated overnight at 37°C. The zymogram was analyzed by iodine staining, and a partial gel was developed using silver staining as contrast material.

To further investigate the abilities of other complex polysaccharides-degradation of strain WPAGA1, arabinogalactan, amylum, sodium alginate, colloidal chitin, carrageenan, carboxymethyl cellulose (CMC), fucoidan, heparin, pectate, porphyran, xylan were used as sole carbon source for the growth of strain WPAGA1 (2 g/L (NH_4_)SO_2_ and 10 g/L of each tested polysaccharide in sea water). The OD_600_ was detected after 24 h of culturing at 28°C and 200 rpm to determine whether strain WPAGA1 could utilize the tested polysaccharide as the carbon source for its growth. Moreover, to directly detect the enzymatic activities of strain WPAGA1 toward different polysaccharides, the secreted extracellular proteins from the WPAGA1 culture were collected and examined for their polysaccharides-degrading activities. In brief, the WPAGA1 strain was cultured at 28°C for 48 h in modified 2216E medium (0.2% yeast extract, 1% peptone, and 0.5% each tested polysaccharide in sea water), and the crude extracellular proteins were prepared as mentioned above. The enzymatic activities of crude extracellular proteins toward various polysaccharides were assayed by detecting the release of the reducing sugar from the corresponding substrates using the 3,5-dinitrosalicylic acid (DNS) method (Miller, 1959) with a slight adaptation. The standard reaction contained 100 μL protein solution and 900 μL of PBS buffer (pH 7.4) containing 0.2% (w/*v*) of each tested polysaccharide. After incubation at 28°C for 10 min, the reaction was stopped by immersion in boiling water for 5 min. The heat-inactivated crude extracellular proteins were used as negative controls. Following this, 250 μL of the reaction solution was mixed with 750 μL of DNS reagent and heated for 10 min in a boiling water bath and then cooled. The absorbance of reducing sugar was measured at 540 nm. Enzyme activity (U) was defined as the amount of enzymes that liberated 1 μmol of reducing sugar per minute under the assay conditions.

### DNA extraction and genome sequencing and assembly

The cells of strain WPAGA1 were collected from the 2 mL culture by centrifugation at 10,000 × g for 10 min. Then, the pellet was washed by sterilized TE buffer twice to remove the residual medium before DNA extraction. The genomic DNA of strain WPAGA1 was extracted using a bacterial DNA kit (OMEGA) and subjected to quality control by agarose gel electrophoresis and quantified by Qubit. The obtained DNA was verified to be of high quality (DNA amount: ≥20 μg and purity: 1.8 ≤ OD260 nm/280 nm ≤ 2.0).

Library construction and sequencing was performed at the Beijing Novogene Bioinformatics Technology Co., Ltd. The genome of strain WPAGA1 was sequenced with massively-parallel-sequencing Illumina technology. Two DNA libraries were constructed, namely, a paired-end library with an insert size of 500 bp and a mate-pair library with an insert size of 5 kb. The 500 bp and 5 kb libraries were sequenced using an Illumina HiSeq2500 apparatus by the PE125 strategy. Quality control of both paired-end and mate-pair reads were performed using an in-house program. After this step, the Illumina PCR adapter reads and low quality reads were filtered. The filtered reads were then assembled by SOAPdenovo (Li et al., 2008, 2010) (http://soap.genomics.org.cn/soapdenovo.html) to generate scaffolds. All reads were used for further gap closure.

### Gene identification

Transfer RNA (tRNA) genes were predicted with a tRNAscan-SE (Lowe and Eddy, 1997), ribosomal RNA (rRNA) genes were predicted with rRNAmmer (Lagesen et al., 2007) and sRNAs were predicted by BLAST against Rfam (Gardner et al., 2009) database. Repetitive sequences were predicted using RepeatMasker (Saha et al., 2008) (http://www.repeatmasker.org/). Tandem repeats were analyzed using Tandem Repeat Finder (Benson, 1999). PHAST (Zhou et al., 2011) was used for prophage prediction (http://phast.wishartlab.com/) and CRISPRFinder (Grissa et al., 2007) was employed for CRISPR (clustered regularly interspaced short palindromic repeat sequences) identification. This Whole-Genome Shotgun project was deposited at the DDBJ/EMBL/GenBank database under the accession number JRYR00000000.

Gene prediction was performed for the *F. pacifica* WPAGA1 genome assembly by GeneMarkS (Besemer et al., 2001) (http://topaz.gatech.edu/) with an integrated model that combines GeneMarkS generated (native) and Heuristic model parameters. A whole-genome BLAST (Altschul et al., 1990) search (*E*-value less than 1e−5, minimal alignment length percentage larger than 40%) was performed against six databases. These databases were KEGG (Kanehisa, 1997; Kanehisa et al., 2004, 2006) (Kyoto Encyclopaedia of Genes and Genomes), COG (Tatusov et al., 1997, 2003) (Clusters of Orthologous Groups), NR (Non-Redundant Protein Database databases), Swiss-Prot (Magrane and UniProt Consortium, 2011), GO (Ashburner et al., 2000) (Gene Ontology) and TrEMBL (Magrane and UniProt Consortium, 2011). A whole-genome BLAST (Altschul et al., 1990) search (E-value less than 1e−5, minimal alignment length percentage larger than 40%) was performed against four databases for pathogenicity and drug resistance analysis. These databases included PHI (Vargas et al., 2012) (Pathogen Host Interactions), VFDB (Chen et al., 2012) (Virulence Factors of Pathogenic Bacteria), ARDB (Liu and Pop, 2009) (Antibiotic Resistance Genes Database), and CAZy (Cantarel et al., 2009) (Carbohydrate-Active enZYmes Database). Secretory proteins were then detected on the genome assembly by SignalP (Petersen et al., 2011). Types I–VII secretion system related proteins were extracted from all the annotation results. Type III secretion system effector proteins were also detected by EffectiveT3 (Jehl et al., 2011). Secondary metabolite gene clusters were predicted by antiSMASH (Medema et al., 2011; Blin et al., 2013). All these annotation files were further combined into one table. Genome overview was created by Circos (Krzywinski et al., 2009) to show annotation information. Searches for the polysaccharide-related metabolic enzymes, including glycoside hydrolases (GHs), glycosyl transferases (GTs), carbohydrate esterases (CEs), polysaccharide lyases (PLs), and auxiliary activities (AAs) were performed manually on the basis of the BLASTP and the CAZy database. These metabolic pathways of polysaccharides were deduced on the basis of such enzymes and the KEGG database.

## Result

### Polysaccharide-degrading ability of *F. pacifica* WPAGA1

**Figure 1:**
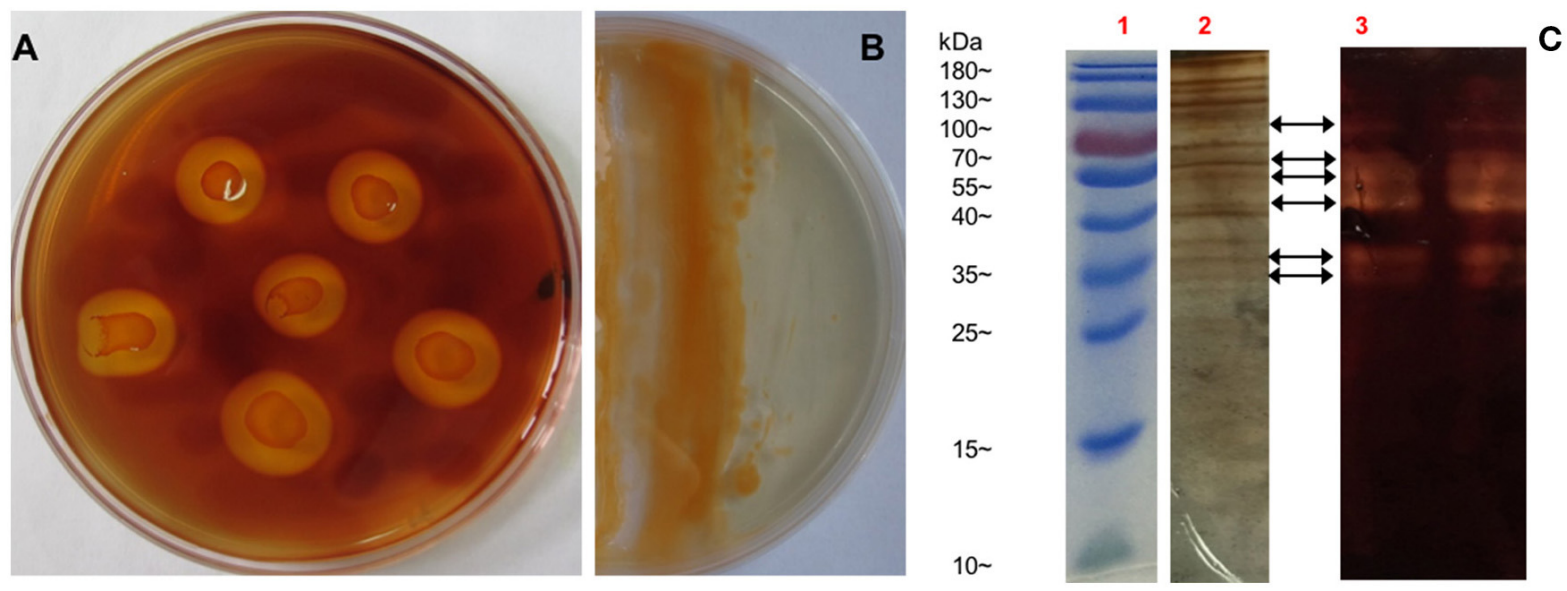
**Agar-degrading ability of ***F. pacifica*** WPAGA1. (A)** *F. pacifica* WPAGA1 cultivated in modified medium (2% agar, 0.2% m/m yeast extract and sea water) for 36 h and afterwards Gram-stained by iodine solution. **(B)** Agar-liquefying properties of *F. pacifica* WPAGA1 on agar medium. **(C)** Zymogram analysis of the extracellular enzyme extract from *F. pacifica* WPAGA1 (arrow). (1) Protein ladder. (2) Sodium dodecyl sulfate–PAGE after silver staining. (3) Agarase activity staining.

As reported, all bacteria belonging to genus *Flammeovirga* can degrade complex polysaccharides, especially the agar that derived from the cell wall of red seaweeds. To investigate the polysaccharide-degrading ability of *F. pacifica* WPAGA1, the agar-degrading profile was first examined. After a 36 h culture in modified 2216E medium plate, strain WPAGA1 spread and formed clear, etched colonies. The plate was stained with iodine solution, and some clear agar degradation zones developed around individual colonies (Figure 1A). The solid medium where strain WPAGA1 was cultured became liquid after 4 days of incubation at 28°C (Figure 1B). Thus, these culture characteristics suggest that WPAGA1 hold potent agarolytic activities.

To further investigate the agar-degrading ability of the strain WPAGA1, extracellular enzymes were extracted from a 48 h culture at 28°C. *F. pacifica* WPAGA1 produced various extracellular proteins (Figure 1C). Moreover, the crude enzymes were run using native PAGE to estimate the agarase components in the extracellular proteins. The iodine staining of the agar gel showed the presence of at least six transparent bands on the developed sheet in Figure 1C. The molecular masses of these agarase bands mainly lay between 35 and 100 kDa. These results show that the strain WPAGA1 harbors a powerful extracellular agarase system for degrading agar.

Besides of the agar, the capabilities of strain WPAGA1 to degrade other complex polysaccharides were further examined. As showed in Table 1, most of the tested polysaccharides could be utilized for the growth of strain WPAGA1 as the sole carbon source. Strain WPAGA1 grew best on alginate (OD_600_ = 1.868), followed by arabinogalactan, carrageenan, porphyran, fucoidan, cellulose, amylum, and xylan. However, strain WPAGA1 could not grow on heparin and pectate. To confirm the above results, the extracellular proteins secreted by strain WPAGA1 were prepared from the culture supernatant, and their enzymatic activities toward the various tested polysaccharides were assayed by the DNS method. As showed in Table 1, strain WPAGA1 exhibited enzymatic activities toward most of the tested polysaccharides except of heparin and pectate, which was consistent with the above results. Therefore, our results showed that the strain WPAGA1 harbors powerful enzymes for degrading various complex polysaccharides, which would enable it to adapt to various environments.

**Table 1:** **The ability of complex polysaccharides-degradation by ***F. pacifica*** WPAGA1**.

**Polysaccharide**	**Culture OD^a^_600_**	**Enzyme activities (U/ml)^b^**
Arabinogalactan	0.525	0.34
Amylum	0.248	1.67
Alginate	1.868	3.45
Chitin	1.729	2.83
Carrageenan	0.517	24.34
Cellulose	0.283	3.56
Fucoidan	0.392	2.49
Heparin	Not grow	–
Pectate or pectin	Not grow	–
Porphyran	0.416	5.93
Xylan	0.171	1.72

a *Culture OD 600 was detected after 24 h of culturing of WPAGA1 strain at 28°C and 200 rpm, which was grown on the tested polysaccharide as the sole carbon source*.

b *The crude extracellular proteins secreted by WPAGA1 strain were prepared from culture supernatant, and their enzymatic activities toward various polysaccharides were detected by the release of the reducing sugar from the corresponding tested polysaccharide using the 3,5-dinitrosalicylic acid (DNS) method*.

### General features and gene annotation of the *F. pacifica* WPAGA1 genome

**Figure 2:**
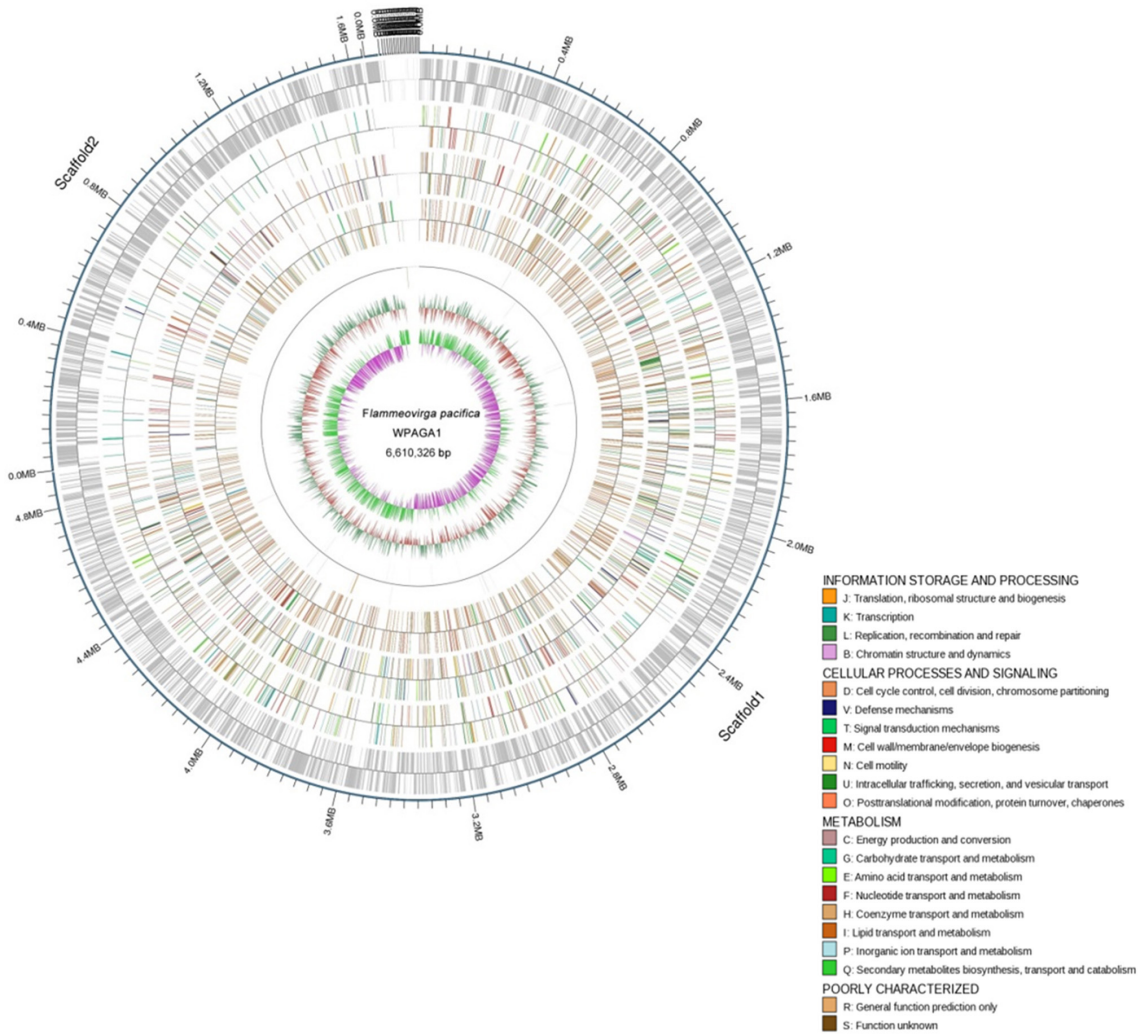
**Physical and genetic maps of the genome of ***F. pacifica*** WPAGA1**. Schematic genome map showing concatenation of all scaffods without certainty of GIs localization is presented. From the outer to the inner concentric circle: circle 1, genomic position in kb; circle 2, predicted protein-coding sequences (CDS) on the forward (out wheel) and reverse (inner wheel) strands; circle 3, annotation results in the GO database; circle 4, annotation results in the KEGG database; circle 5, annotation results in the COG database; circle 6, ncRNA region; circle 7, G+C content showing deviations from the average (32.89%); circle 8, GC skew.

To further explore the genetic basis for the ability of complex-polysaccharide degradation, the genome of *F. pacifica* WPAGA1 was sequenced. By using an Illumina HiSeq2500 apparatus and PE125 genome sequencing strategy, we constructed the following two DNA libraries: a paired-end library with an insert size of 500 bp and a mate-pair library with an insert size of 5 kb. As a result, a total of 625 Mb (500 bp) and 1,317 Mb (5 kb) reads were obtained. The generated sequences were assembled into 65 contigs with an *N*_50_ length of 445,839 bps, and these contigs were assembled into 21 scaffolds. Accordingly, the fine genome of *F. pacifica* WPAGA1 consists of 6,610,326 bases with 32.89% G+C content, 333 interspersed repeated sequences, 229 tandem repeats finder, 83 tRNA genes, 5 rRNAs (two 5S rRNA, two 16S rRNA, and one 23S rRNA), 8 genomics islands and especially, 5 questionable CRISPRs (Figure 2, Table 2). A total of 5,036 ORFs, which account for 87.37% of the total nucleotides, were obtained. Amongst these genes, 3,189 protein-coding sequences (63.32% of the total) were annotated and identified by NR search with GenBank sequences. In addition, 957, 1,660, 1,090, 1,487 and 2,983 proteins were functionally annotated from UniProtKB/Swiss-Prot, KEGG, COG, GO, and UniProtKB/TrEMBL databases, respectively. The detailed results are shown in Tables 2, 3.

**Table 2:** **General features of the ***F. pacifica*** WPAGA1 genome**.

**Feature**	
Size (bp)	6,610,326
G+C content (%)	32.89
Coding sequences	5,577,114
Genes number	5,036
Gene average length (bp)	1,147
% Gene lengthG~enome	87.37
RNA elements	
rRNA	5
tRNA	83
Genomic islands	
GL number	8
GL total length (bp)	133,208
Average length (bp)	16,651
CRISPR	
Potential gene number	5

**Table 3:** **Gene annotation in databases**.

**Database**	**Number**
NR	3,189
SwissProt	957
KEGG	1,660
COG	1,090
GO	1,487
TrEMBL	2,983

### Genomic islands and genetic elements

The *F. pacifica* WPAGA1 genome contains a number of regions, such as genomic island, with atypical GC content. Eight genomic islands were found, and the average length was 16,651 bp. Genomic island (GIs) 1 (GIs1, 2,634,781 bp to 2,663,274 bp, Scaffold1) comprises a cluster of 29 genes with products predicted to be involved in tRNA-modifying proteins. Interestingly, three genes that encode proteins showed the highest similarity to protein HigA, phage antirepressor protein and plasmid maintenance system killer protein belonging to phage genes or plasmid gene sequences. GIs 4 (3,802,724–3,584,014 bp, Scaffold1) carries a gene cluster for Clp protease ClpC. GIs 7 (314,546–333,456 bp, Scaffold2) comprises a series of genes with products predicted to be involved in stress-resistant proteins, such as the acriflavin resistance protein, tellurium resistance protein, beta-lactamase and arsenic resistance protein ArsB. Some compound genes, such as the cobalamin synthase gene, spore coat protein gene, ABC transporter genes, transposase genes and rich phosphatase genes were found in these genomic islands.

The *F. pacifica* WPAGA1 genome harbors a considerable number of genetic elements, such as 333 interspersed nuclear elements and 88 non-coding RNA. Especially, five regions of nucleotide sequences with products predicted to be CRISPR-associated genes were found. These CRISPR-associated genes show that *F. pacifica* WPAGA1 was invaded by exogenous DNA or RNA, such as phage.

### Genetic basis of polysaccharide degradation in *F. pacifica* WPAGA1

**Figure 3:**
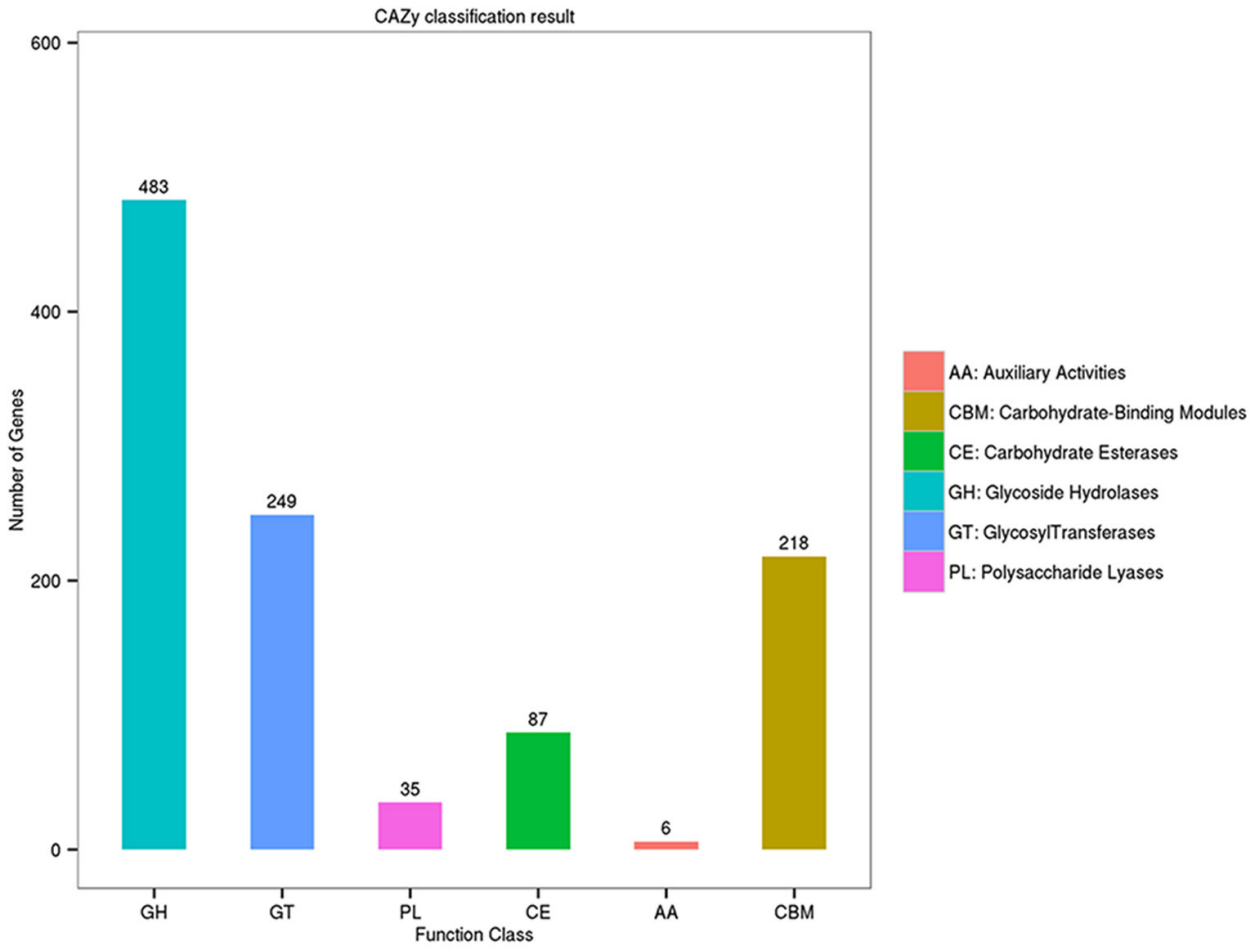
**Annotation of carbohydrate-related genes in the Carbohydrate-active Enzymes (CAZy) database**.

A distinguishing feature of the *Flammeovirga* genus is the capability to produce a series of enzymes for polysaccharide hydrolysis or lysis. To search for genes related to polysaccharide-degrading enzymes in the genome of *F. pacifica* WPAGA1, the CAZy database was used for annotating carbohydrate-related genes. A total of 1,022 carbohydrate-related genes were annotated by the CAZy database (Figure 3). Amongst these genes, 483 genes belong to glycoside hydrolases (GHs), followed by 249 genes identified with glycosyl transferases (GTs) and 87, 35, and 6 genes that belong to carbohydrate esterases (CEs), polysaccharide lyases (PLs) and auxiliary activities (AAs), respectively. Meanwhile, 218 proteins contain carbohydrate-binding modules (CBM) defined as contiguous amino-acid sequences within a carbohydrate-active enzyme with a discreet fold having carbohydrate-binding activity. Bacteria catalyze polysaccharide depolymerization by secreting an array of lyases or glycosidase that generate oligosaccharides or monosaccharides. These bacteria then undergo a complex series of random or regular cleavage reactions. The major components of polysaccharide-degrading enzymes are GHs and PLs. Simultaneously, 483 putative GH proteins and 35 putative PL proteins were found in the *F. pacifica* WPAGA1 genome (Figure 3). These masses of carbohydrate-active proteins comprise a complex system for carbohydrate catabolism in *F. pacifica* WPAGA1. We next investigated the detailed polysaccharide-degrading pathways in the WPAGA1 strain with respect to various polysaccharides on the basis of genomic analysis. Detailed gene information related to polysaccharide metabolism is shown in Table 4 and Table S1.

**Table 4:** **Polysaccharide-degrading enzymes in the ***F. pacifica*** WPAGA1 genome**.

**Polysaccharide**	**Catabolic enzymes**	**Enzyme family**	**No. of enzymes**	**Products**
Agarose	β-agarase	GH16	1	Neoagarotetraose, neoagarohexaose, neoagarobiose
		GH50	1	Neoagarobiose
		GH86	11	Neoagarotetraose, neoagarohexaose, neoagarobiose
Arabinogalactan	Endo-1,4-beta-galactosidase	GH53	1	tetrasaccharide
Amylum	α-Amylase	GH13	7	Dextrin, maltodextrin, maltose, α-D-glucose
	β-Amylase	GH14	1	maltose
Alginate	Alginate lyase	PL7	4	Oligosaccharides^a^
Chitin	Chitinase	GH18	9	Chitooligosaccharide^b^
	Chitin deacetylase	CE4	2	Chitosan
Carrageenan	κ- Carrageenase	GH16	1	Neocarrabiose-sulfate, neocarratetraose-sulfate
	ι-Carrageenase	GH82	5	ι-Neocarratetraose sulfate, ι-neocarrahexaose sulfate
	λ-Carrageenase	PL11	2	Tetrasaccharide
Cellulose	Cellulose	GH9	2	Cellobiose
Fucoidan	α-L-Fucosidase	GH29G~H95	18	L-Fucose
Heparin	Heparinase	PL17	1	Oligosaccharides^c^
Pectate or pectin	Pectin lyase	PL1	2	Oligosaccharides^d^
	Pectate lyase	PL1	2	Oligosaccharides^d^
Porphyran	β-Porphyranase	GH16	1	α-L-Galactopyranose-6-sulfate-(1→3)-β-D-galactose,
Rhamnogalacturonan	Rhamnogalacturonan lyase	PL11	1	Oligosaccharides^e^
Xylan	Endo-1,4-beta-xylanase	GH8	1	Oligosaccharides^f^
	Endo-1,4-beta-xylanase	GH10	2	Oligosaccharides^g^
	Endo-1,3-beta-xylanase	GH26	3	Xylobiose, xylotriose, and xylotetraose
	Xylan 1,4-beta-xylosidase	GH39	3	D-xylose
	Xylan 1,4-beta-xylosidase	GH43	1	D-xylose

*Oligosaccharides^a^: oligosaccharides with a complex copolymer of α-L-guluronate and its C5 epimer β-D-mannuronate; Chitooligosaccharide^b^: random hydrolysis of N-acetyl-β-D-glucosaminide (1→4)-β-linkages in chitin and chitodextrins; Oligosaccharides^c^: oligosaccharides with terminal 4-deoxy-α-D-gluc-4-enuronosyl groups at their non-reducing ends; Oligosaccharides^d^: oligosaccharides with 4-deoxy-6-O-methyl-α-D-galact-4-enuronosyl groups at their non-reducing ends; Oligosaccharides^e^: Oligosaccharides with L-rhamnopyranose at the reducing end and 4-deoxy-4,5-unsaturated D-galactopyranosyluronic acid at the non-reducing end; Oligosaccharides^f^, Oligosaccharides^g^: endohydrolysis of (1→4)-β-D-xylosidic linkages in xylans to generate oligosaccharides*.

#### Agar-degrading pathway

**Figure 4:**
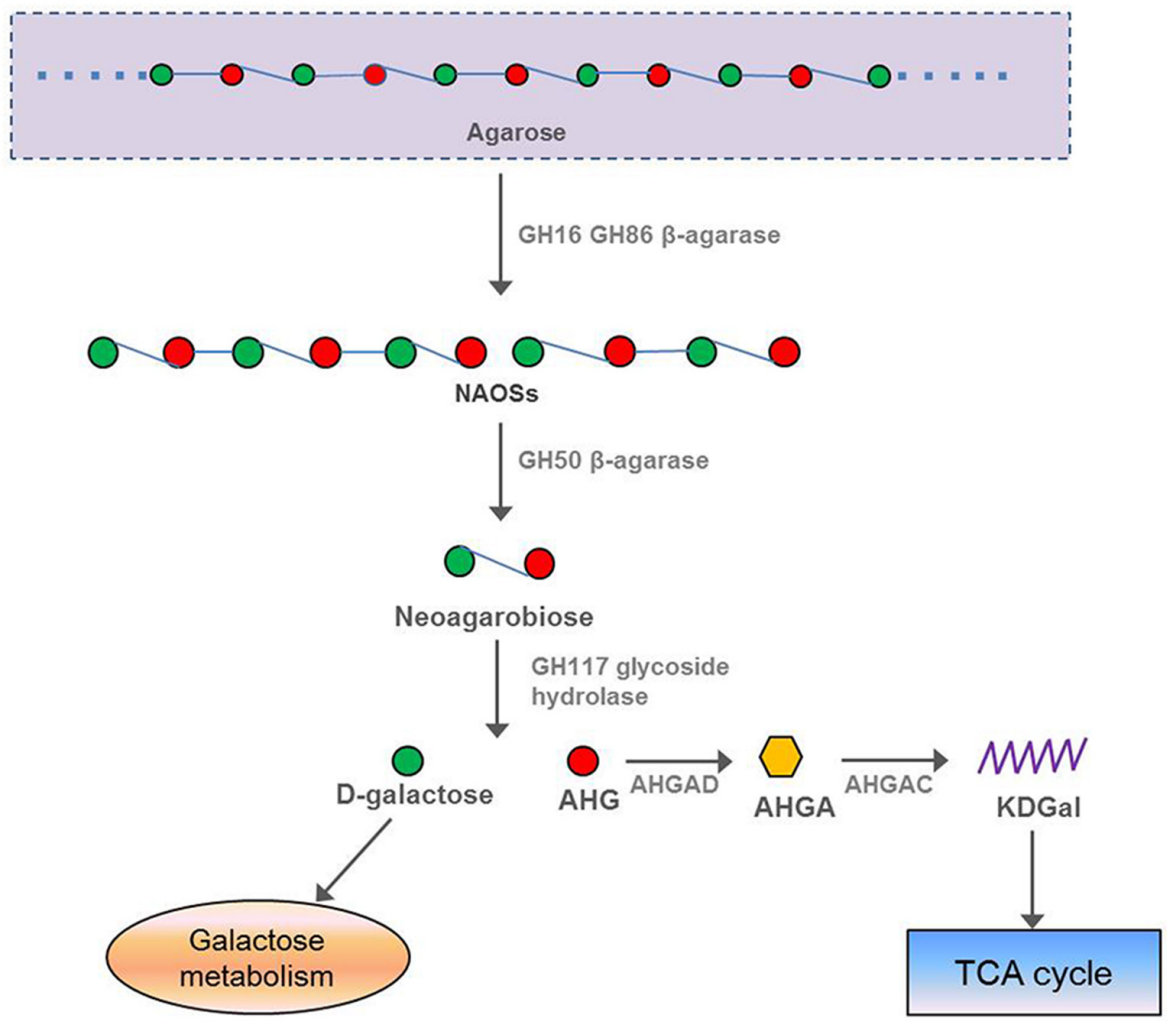
**Putative pathway of agar degradation in ***F. pacifica*** WPAGA1**. Agarose is hydrolyzed into NAOSs by GH16-dependent or GH86-dependent enzymes and then further degraded into neoagarobiose by GH50-dependent agarase. Neoagarobiose is then hydrolyzed by the GH117 family glycoside hydrolase into D-galactose and AHG. Finally, D-galactose enters the galactose metabolic pathway, and AHGAD and AHGAC catalyze AHG conversion to KDGal, which is utilized by the TCA cycle. NAOSs, neoagarooligosaccharides; AHG, 3,6-anhydro-L-galactose; AHGAD, AHG dehydrogenase; AHGA, 3,6-anhydrogalactonate; AHGAC, AHGA cycloisomerase; KDGal, 2-keto-3-deoxy-galactonate.

In our study, 13 β-agarase genes were found in the *F. pacifica* WPAGA1 genome, whereas the α-agarase genes were not found. The protein FlaGM004591, which encodes a GH16 β-agarase, exhibited a high amino-acid similarity (87%) to the β-agarase in *F. yaeyamensis*, which has been reported to hydrolyse agarose into neoagarooligosaccharides (NAOSs) through the endolytic cleavage of the β-1,4-linkages of agarose. Another agarase belonging to GH50 was encoded by *F. pacifica* WPAGA1 ORF FlaGM002660, which showed a low amino-acid similarity (40.6%) to the β-agarase in *Coraliomargarita akajimensis* DSM 45221. Some studies reported that NAOSs are further degraded into neoagarobiose by the GH50-dependent β-agarase (Kim et al., 2010; Zhou et al., 2015). Finally, the glycoside hydrolase belonging to the GH117 family hydrolyses neoagarobiose into D-galactose and AHG (Ha et al., 2011; Hehemann et al., 2012; Pluvinage et al., 2013). Similarly, protein FlaGM004900, which exhibited a high amino-acid similarity (91.8%) to the GH family 117 glycoside hydrolase, is also present in the *F. pacifica* WPAGA1 genome. D-galactose is known to be next utilized by the galactose metabolic pathway, but the intact metabolic pathways of AHG degradation was unclear in the past. Recently, two key metabolic intermediates of AHG, namely, 3,6-anhydrogalactonate (AHGA) and 2-keto-3-deoxy-galactonate (KDGal), and their corresponding enzymes, namely, AHG dehydrogenase (AHGAD) and AHGA cycloisomerase (AHGAC), were discovered for the first time (Choi et al., 2011, 2013). The proposed catabolic pathway of AHG begins with the oxidation of an aldehyde group of AHG to AHGA by AHG dehydrogenase, and AHGA is then isomerized by AHGA cycloisomerase to form KDGal, which is a common metabolic intermediate for microorganisms (Choi et al., 2011, 2013; Yun et al., 2015). Importantly, *F. pacifica* WPAGA1 also encodes a AHGA cycloisomerase by FlaGM004986, which showed 73% amino-acid similarity to AHGA cycloisomerase in *Vibrio* sp. EJY3, and two AHG dehydrogenases by FlaGM004985 and FlaGM004649, which exhibited 64% and 44% amino-acid similarities, respectively, to AHGA cycloisomerase in *Vibrio* sp. EJY3. In addition, 11 β-agarase, which belongs to the GH86 family and hydrolyses agarose into NAOSs, are harbored by the *F. pacifica* WPAGA1 genome. Thus, the agar metabolic pathway of *F. pacifica* WPAGA1 was deduced on the basis of genomic data. In particular, GH16-dependent or GH86-dependent β-agarase initially hydrolyses agarose into NAOSs and are then further degraded into neoagarobiose by GH50-dependent agarose, followed by the GH117 family glycoside hydrolase, which hydrolyses neoagarobiose into D-galactose and AHG. Finally, D-galactose enters into the galactose metabolic pathway, and AHG is catalyzed by AHG dehydrogenase and AHGA cycloisomerase to form KDGal, which is utilized by the TCA cycle. Thus, *F. pacifica* WPAGA1 possesses the complete pathway for the use of agarose (Figure 4).

#### Other polysaccharide-degrading enzymes

##### Carrageenan-degrading enzymes

Carrageenan, which widely exists in the marine environment, are a family of linear sulphated polysaccharides extracted from red edible seaweeds (Agar FAO and Manual, 2011). Three main varieties of carrageenan, namely, κ-carrageenan, ι-carrageenan, and λ- carrageenan, exist. These polysaccharides differ in their degree of sulphation. Therefore, the three types of enzymes (κ-carrageenase [EC 3.2.1.83], ι-carrageenase [EC 3.2.1.157], and λ-carrageenase [EC 3.2.1.162]) are used to catalyze carrageenan degradation in the wild. A total of 8 carrageenases were contained in *F. pacifica* WPAGA1 (Table 4). The protein FlaGM003973 encoded by *F. pacifica* WPAGA1 exhibits 41% amino-acid similarity to the identified κ-carrageenase in *Rhodopirellula baltica* strains, which has been classified within the GH family 16. The κ-carrageenase, which belongs to the GH family 16, endohydrolyses the (1→4)-β-D-linkages between the D-galactose 4-sulfate and 3,6-anhydro-D-galactose in κ-carrageenans, and the main products of hydrolysis are neocarrabiose-sulfate and neocarratetraose-sulfate. A second carrageenan hydrolase system is specified by FlaGM004000, FlaGM004113, FlaGM004431, FlaGM004624, and FlaGM004753, encoding the same GH82 enzyme ι-carrageenase, which endohydrolyses the (1→4)-β-D-linkages between D-galactose 4-sulfate and 3,6-anhydro-D-galactose-2-sulfphate in ι-carrageenans. The main products of hydrolysis are ι-neocarratetraose sulfate and ι-neocarrahexaose sulfate. These five proteins were predicted as enzyme ι-carrageenase from *Zobellia galactanivorans*, but amino-acid similarities to the enzyme were dissimilar and showed that these proteins may differ in hydrolytic enzyme activity. Another carrageenan hydrolase system in *F. pacifica* WPAGA1 were designated as FlaGM003999 and FlaGM004436 and exhibited low percentage (46.4 and 45.7%, respectively) similarities to the PL11 λ-carrageenase in *Pseudoalteromonas* sp. CL19. This hydrolase system could endohydrolyse the (1→4)-β-linkages in the λ-carrageenan backbone, resulting in a tetrasaccharide.

##### Alginate-degrading enzymes

*F. pacifica* WPAGA1 can degrade alginate, a copolymer of α-L-guluronate (G) and its C5 epimer β-D-mannuronate (M) arranged as homopolymeric G blocks, M blocks, alternating GM, or random heteropolymeric G/M stretches. This enzyme is found in great abundance as part of the cell wall and intracellular material in brown seaweed (Phaeophyceae) (Rowley et al., 1999). Alginate lyases, the main alginate-degrading enzyme, was isolated from various marine bacteria. Meanwhile, the *F. pacifica* WPAGA1 genome harbors four alginate lyase genes and belong to PL family 7 that showed the *F. pacifica* WPAGA1 could degrade alginate (Table 4). The protein FlaGM002034 exhibited a 32% amino-acid similarity to the well-characterized alginate degradation enzyme in *Niastella koreensis* GR20-10 strain. The putative protein FlaGM002592 also shows high amino-acid identity (67%) with alginate lyase of *Z. galactanivorans*. Other two putative proteins involved in the alginate degradation include FlaGM002039 and FlaGM004227, which presented low and high percentages, respectively, of amino-acid similarity to the alginate lyases in *Flammeovirga* sp. MY04. Previous studies showed that alginate lyases catalyze alginate degradation depending on alginate type, as well as substrate specificity. Equally, four putative proteins encoded by the *F. pacifica* WPAGA1 genome showed different amino-acid similarities to various alginate lyases. This result indicates that *F. pacifica* WPAGA1 may degrade various alginate types.

##### Xylan-degrading enzymes

*F. pacifica* WPAGA1 can degrade another polysaccharide, specifically, xylan, which is a group of hemicelluloses found in plant cell walls and some algae (Ebringerová and Heinze, 2000). This polysaccharide is derived from xylose units (Ebringerová and Heinze, 2000). Some bacteria were reported as degraders of xylan and called xylanases, which belong to the GH system. Ten putative xylanases, including three putative endo-1,4-β-xylanases (EC 3.2.1.8), three putative endo-1,3-β-xylanases (EC 3.2.1.32), and four putative xylan 1,4-β-xylosidases (EC 3.2.1.37), exist in *F. pacifica* WPAGA1. The protein FlaGM001213 exhibited a low amino-acid identity with endo-1,4-β-xylanases of *Cytophaga hutchinsonii* ATCC 33406, which were grouped within the GH8 family. This enzyme endohydrolyses (1→4)-β-D-xylosidic linkages in xylans to produce oligosaccharides. The other two endo-1,4-β-xylanases, proteins FlaGM004517 and FlaGM004522, which belong to GH10, displayed 42 and 53% amino-acid similarities, respectively, to the xylanases in *Flavobacterium johnsoniae* UW101 and *Pseudopedobacter saltans* DSM 12145, respectively. Other proteins involved in xylan degradation, namely, xylan 1,4-β-xylosidase and endo-1,3-β-xylanase, showed different percentage similarities with the orthologs listed in Table 4 and Table S1.

##### Cellulose-degrading enzymes

**Figure 5:**
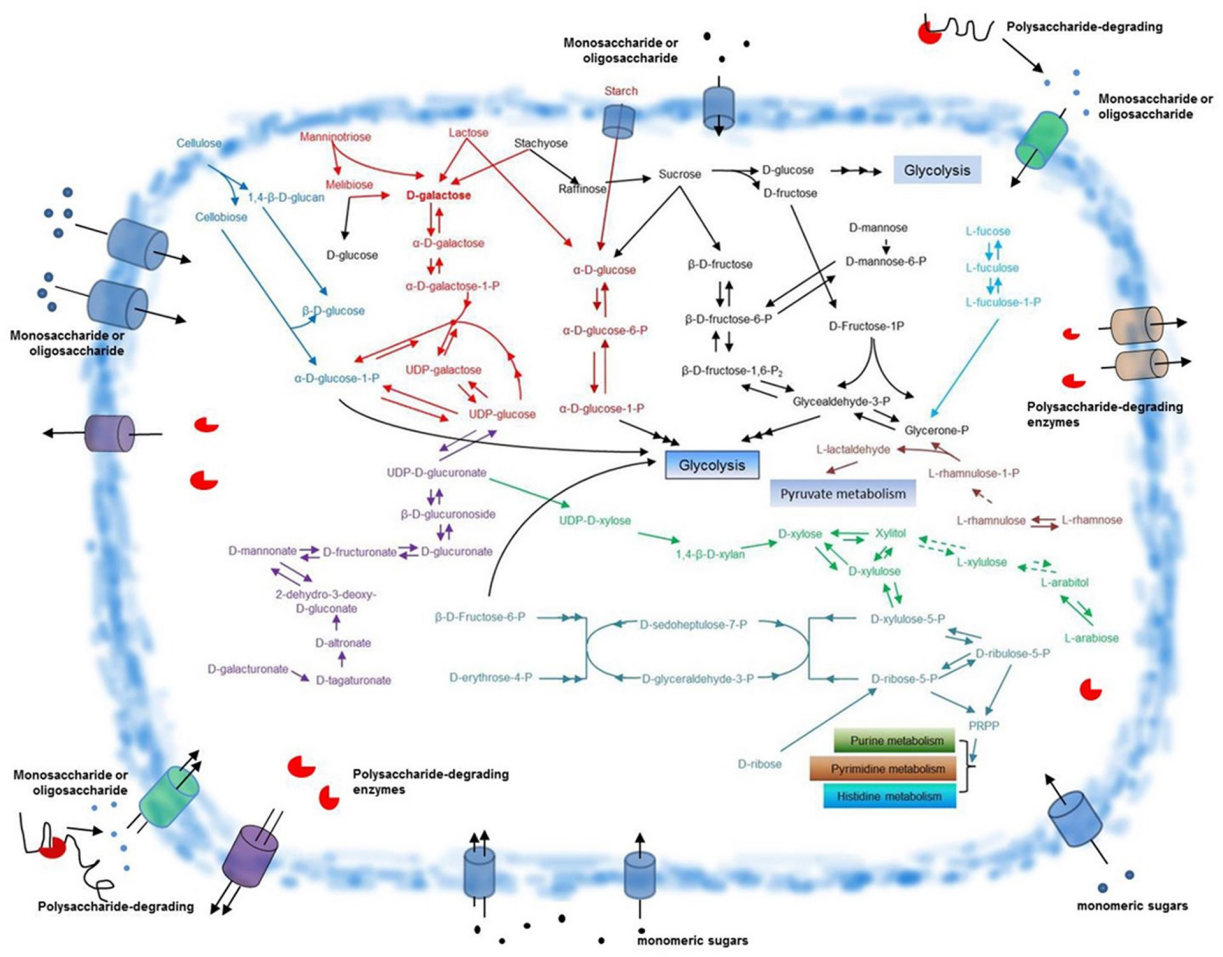
**Schematic overview of glycometabolism in ***F. pacifica*** WPAGA1**. Polysaccharide-degrading enzymes are secreted extracellularly and catalyze the degradation of complex polysaccharides to generate various monomeric sugars or oligosaccharides. These sugars are transported into the *F. pacifica* WPAGA1 cell and are utilized by complex glycometabolic pathways. The predicted pathway for D-galactose metabolism is depicted in red, cellulose metabolism in blue, starch metabolism in dark red, D-galacturonate metabolism in violet, D-ribose metabolism in aqua green, L-arabiose metabolism in green, L-rhamnose metabolism in light red, L-fucose metabolism in light blue and other sugar metabolic pathways in black. Dotted arrows denote corresponding enzymes that are not annotated; points signify monosaccharides or oligosaccharides. PRPP, 5′-phospho-alpha-D-ribose1-diphosphate.

*F. pacifica* WPAGA1, like most bacteria, utilizes glucose as the main energy source. By contrast, unlike other bacteria, *F. pacifica* WPAGA1 obtains additional sources of glucose from its complex polysaccharide-degrading metabolic pathways. Cellulose is degraded by *F. pacifica* WPAGA1 to generate glucose (Table 4 and Figure 5), but in nature, such process generally occurs in fungi. In *F. pacifica* WPAGA1, cellulose is initially converted to cellobiose and 1,4-β-D-glucan by a putative cellulase (EC 3.2.1.4, FlaGM000759). 1,4-β-D-Glucan is further transformed into β-D-glucose, which could be directly utilized for the glycolytic pathway via the richly putative beta-glucosidase (EC 3.2.1.21) encoded by *F. pacifica* WPAGA1. Cellobiose is degraded by a putative cellobiose phosphorylase (EC 2.4.1.20, FlaGM001118) to generate α-D-glucose-1-P and β-D-glucose. Furthermore, these two glucose molecules could directly enter the glycolytic pathway. Thus, *F. pacifica* WPAGA1 utilizes cellulose by this metabolic pathway. Another important source of glucose is starch degradation. In *F. pacifica* WPAGA1, starch could be degraded to α-D-glucose by a putative glucan 1,4-α-glucosidase (EC 3.2.1.3, FlaGM002135) and hydrolyzed by other enzymes (Figure S4). Notably, some putative polysaccharide deacetylases, such as FlaGM000887 or FlaGM000981, are present in *F. pacifica* WPAGA1. The presence of these enzymes indicates that *F. pacifica* WPAGA1 could deacetylate polysaccharides, such as chitin, by polysaccharide deacetylase, and the resultant products are hydrolyzed by GH.

Besides the enzymes responsible for degrading these above-mentioned polysaccharides, numerous other polysaccharide-degrading enzymes were predicted from the *F. pacifica* WPAGA1 genome, including those enzymes that can catalyze the degradation of amylum, chitin, fucoidan, heparin, pectate, porphyran, rhamnogalacturonan, and arabinogalactan (Table 4). The higher versatility and wider spectrum for carbohydrate degradation clearly provides *F. pacifica* WPAGA1 a competitive advantage over other members of the polysaccharide-degrading marine microbial communities.

### Glycometabolism involved in polysaccharide usage in *F. pacifica* WPAGA1

As described above, substantial amounts of polysaccharide-degrading enzymes are found in *F. pacifica* WPAGA1. Abundant sugar source is produced by the polysaccharide-degrading enzyme system. Our data show that the pentose phosphate pathway (PPP), pentose and glucoronate interconversion, fructose and mannose metabolism, galactose metabolism and starch and sucrose metabolism constitute the main pathways that enable *F. pacifica* WPAGA1 to utilize these sugars for growth. We deduced a schematic of glycometabolism in *F. pacifica* WPAGA1 (Figure 5).

#### D-galacturonate metabolic pathway

D-galacturonate is the main degradation product of pectate or pectin, and genome sequences showed that the pentose and glucoronate interconversion pathway (Figure S1) is involved in the D-galacturonate usage of *F. pacifica* WPAGA1. In Figure 5 (in purple), D-galacturonate is transformed into D-tagaturonate via a putative glucuronate isomerase (EC 5.3.1.12, FlaGM00472). Then, D-tagaturonate is transformed into D-altronate by tagaturonate reductase (EC 1.1.1.58, FlaGM001960). Afterward, a putative altronate dehydratase (EC 4.2.1.7, FlaGM001961) catalyzes D-altronate transformation into 2-dehydro-3-deoxy-D-gluconate, which is a kind of frequent intermediate metabolite. Next, the putative mannonate dehydratase (EC 4.2.1.8, FlaGM004723) catalyzes the transformation of 2-dehydro-3-deoxy-D-gluconate into D-mannonate. Subsequently, D-fructuronate is further produced via a putative mannonate oxidoreductase (EC 1.1.1.57, FlaGM004724). D-fructuronate is transformed into D-glucuronate by a putative glucuronate isomerase (EC 5.3.1.12, FlaGM004721) and then forms β-D-glucuronoside via putative β-glucuronidases (EC 3.2.1.31, FlaGM000194, FlaGM003679, and FlaGM004982). β-D-glucuronoside is converted to UDP-D-glucuronate by glucuronosyltransferase, which is not found in genome sequences (green dashed arrows). Finally, UDP–glucose is produced via a putative UDP–glucose 6-dehydrogenase (EC 1.1.1.22, FlaGM000121, FlaGM000125). UDP–glucose is further transformed into α-D-glucose-1-P, which directly enters glycolysis or forms UDP-D-xylose, which enters the PPP.

#### Hexose metabolic pathway

The hexose fucose, which is a hexose deoxysugar usually found in N-linked glycans and the fundamental subunit of the fucoidan polysaccharide, is also utilized by *F. pacifica* WPAGA1. The metabolic pathway involved in fucose utilization could be deduced from the genome sequences (Figure 5, in light blue). Firstly, L-fucose is transformed into L-fuculose via the putative L-fucose isomerase (EC 5.3.1.25, FlaGM003966). This process is followed by the phosphorylation of putative L-fuculokinase (EC 2.7.1.51, FlaGM003967) and the formation of L-fuculose-1-P. Then, L-fuculose-1-P is transformed into glycerone-P by the putative L-fuculose-phosphate aldolase (EC 4.1.2.17, FlaGM004648). Glycerone-P next forms glycealdehyde-3-P, which enters glycolysis through the action of a putative triose-phosphate isomerase (EC 5.3.1.1, FlaGM002926). The other hexoses, such as mannose and rhamnose (in black), could also be utilized by *F. pacifica* WPAGA1. Similarly, L-rhamnose is converted to L-rhamnulose by a putative L-rhamnose isomerase (EC 5.3.1.14, FlaGM004021) and is phosphorylated by rhamnulokinase, which is not found in the genome sequences. Then, L-rhamnulose-1-P is transformed into glycerone-P and L-lactaldehyde by rhamnulose-1-phosphate aldolase (EC 4.1.2.19). Glycerone-P is utilized by the glycolytic pathway, and L-lactaldehyde is further metabolized by pyruvate metabolism. Another hexose, mannose, which is a sugar monomer of the aldohexose series of carbohydrates, could also be metabolized by *F. pacifica* WPAGA1. Firstly, D-mannose is phosphorylated by hexokinase (EC 2.7.1.1) to generate D-mannose-6-phosphate, but hexokinase has not been annotated in the genome sequences. D-mannose-6-phosphate is converted to β-D-fructose-6-P by the enzyme phosphomannose isomerase (EC 5.3.1.8, FlaGM003807 and FlaGM003955) and then enters the glycolytic pathway. The metabolic pathways of the above-mentioned three hexoses are involved in fructose and mannose metabolism, and the detailed fructose and mannose metabolic pathways in *F. pacifica* WPAGA1 is shown in Figure S2.

#### Pentose metabolic pathway

Except for hexose, pentose, which is hardly utilized in many bacteria, could also be metabolized by *F. pacifica* WPAGA1. Arabinose is an aldopentose, that is, a monosaccharide containing five carbon atoms and an aldehyde (CHO) functional group. In nature, this monosaccharide combines with another monosaccharide to form a heterosaccharide. For biosynthetic reasons, most saccharides are almost always more abundant in nature as the “D”-form than the “L”-form. However, L-arabinose can also be metabolized in *F. pacifica* WPAGA1 as revealed by genome prediction. According to the genome sequence data of *F. pacifica* WPAGA1, the metabolic pathway involved in L-arabinose (in green) biosynthesis was predicted. L-arabinose is transformed into L-arabitol via the putative aldehyde reductase (EC 1.1.1.21, FlaGM003171 and FlaGM003572), followed by the formation of L-xylulose by L-arabinitol 4-dehydrogenase (EC 1.1.1.12), which has not been annotated in this genome sequences. L-xylulose is further transformed into xylitol by L-xylulose reductase (EC 1.1.1.10), which was not annotated, and finally, xylitol forms D-xylose by the putative aldehyde reductase (EC 1.1.1.21, FlaGM003171 and FlaGM003572) or is converted to D-xylulose by the D-xylulose reductase (EC 1.1.1.9, FlaGM001965). These two monosaccharides both enter the PPP. The other two pentoses, D-ribose (in aqua green) and D-xylose (in green), could also be metabolized in *F. pacifica* WPAGA1. D-ribose or D-xylose must be phosphorylated by the cell before these sugars can be used. Thus, the corresponding phosphatase is the key step in the use of D-ribose and D-xylose in the cell. Consistent with the prediction, the putative ribokinase (EC 2.7.1.15, FlaGM001539), which phosphorylated D-ribose to generate D-ribose-5-P was annotated in the genome sequences. Meanwhile, D-xylose must be transformed into D-xylulose by the putative xylose isomerase (EC 5.3.1.5, FlaGM003094). Then, D-xylulose is phosphorylated by the putative xylulokinase (EC 2.7.1.17, FlaGM003096) to generate D-xylulose-5-P. Subsequently, D-ribose-5-P and D-xylulose-5-P are simultaneously converted to D-sedoheptulose-7-P and D-glyceraldehyde-3-P by the putative transketolase (EC 2.2.1.1, FlaGM000854, FlaGM000892, FlaGM001423, and FlaGM003098) and are then converted to D-erythrose-4-P and β-D-fructose-6-P by the putative transaldolase (EC 2.2.1.2, FlaGM000683, FlaGM003097 and FlaGM003449). Finally, β-D-fructose-6-P enters the glycolytic pathway. The metabolic pathways of these three pentoses in *F. pacifica* WPAGA1 are involved in the pentose and glucoronate interconversion pathway and PPP. The detailed information is presented in Figures S2, S3.

#### D-galactose metabolic pathway

*F. pacifica* WPAGA1 degrades many polysaccharides, such as agar, carrageenan or porphyran, to generate D-galactose. This observation shows that D-galactose is the important carbon source for *F. pacifica* WPAGA1, and the genome contains genetic information for encoding 49 putative carbohydrate-active enzymes involved in galactose metabolism. Besides the main source of D-galactose from polysaccharide, manninotriose, lactose and stachyose could also produce D-galactose (in red) (Figure 5). In Figure 5, D-galactose is initially converted to α-D-galactose by the putative aldose 1-epimerase (EC 5.1.3.3, FlaGM004262, FlaGM004568, FlaGM004894, and FlaGM004987), then α-D-galactose is phosphorylated by the putative galactokinase (EC 2.7.1.6, FlaGM004565) to generate α-D-galactose-1-P. Afterwards, α-D-galactose-1-P and UDP–glucose form α-D-glucose-1-P and UDP-galactose through the putative hexose-1-phosphate uridylyltransferase (EC 2.7.7.12, FlaGM004566). α-D-glucose-1-P could be utilized by the glycolytic pathway, whereas UDP–galactose is further transformed to UDP–glucose by the putative UDP–glucose 4-epimerase (EC 5.1.3.2, FlaGM002305, FlaGM002421, FlaGM003854, and FlaGM004567). Thus, *F. pacifica* WPAGA1 can metabolize abundant galactose by degrading polysaccharide through its complex enzyme system. Furthermore, the entire galactose metabolic pathway (Figure S5) involved in D-galactose utilization could be deduced from the genome sequence. Results show that *F. pacifica* WPAGA1 possesses a competitive advantage over other members of marine microbial communities in the galactose-rich marine environment.

Besides the enzymes mentioned above, some carbohydrate-active enzymes are predicted in the *F. pacifica* WPAGA1 genome, such as enzymes involved in the inositol phosphate metabolism, pyruvate metabolism and ascorbate and aldarate metabolism. However, these predicted pathways are incomplete or partial because of the unpredicted key enzymes in the genome.

## Discussion

Previous studies showed that *Flammeovirga* bacteria can degrade various polysaccharides, especially agar, a typical polysaccharide derived from red seaweeds (Hou et al., 2015; Zhou et al., 2015). *Flammeovirga* bacteria have been proposed to possess a characteristic genomic basis for complex-polysaccharide usage and could thus adapt and survive in the marine environment. However, the genome information of *Flammeovirga* bacteria is still largely unknown. In our study, a high-quality fine genome of *F. pacifica* WPAGA1 was generated. Genomic sequence analysis identified 5,036 ORFs, which exhibited relatively low similarity to previously reported genes in other bacterial species, including other species of *Flammeovirga*. The genome size of *F. pacifica* WPAGA1 is 6,610,326 bp, which includes eight genomic islands, abundant interspersed nuclear elements and the other genomic elements. Genomic islands are specific regions with atypical GC contents in genome. Many previous studies have showed that rich resistance genes, such as a variety of antibiotic genes, are harbored in these regions (Boyd et al., 2001, 2002; Ito et al., 2003). So these regions are beneficial to improve the viability of bacteria. And the unusual nucleotide compositions of genomic islands may indicate horizontal gene transfer or may have originated from numerous factors, such as mutation bias. The genomic G+C content of *F. pacifica* WPAGA1 is lower than those of most bacteria. This information potentially indicates the species' novel and specific metabolic and survival strategy in deep-sea environments. Generally, high G+C genomic content is shown in adverse survival environment. Meanwhile, a low G+C content reflects a mild survival environment (Galtier and Lobry, 1997). Interestingly, *F. pacifica* WPAGA1 was isolated from deep-sea sediment (Xu et al., 2012), which is inconsistent with our present genomic sequence result, that is, the low genomic G+C content of *F. pacifica* WPAGA1. Meanwhile, some studies reported a *Flammeovirga* species isolated from coastal sediments (Han et al., 2012), which comprise a relatively mild environment. Thus, we propose that *F. pacifica* WPAGA1 may originate from shallow sea. Furthermore, a large number of carbohydrate-active enzyme encoding genes were found in the *F. pacifica* WPAGA1 genome, especially abundant genes involved in polysaccharide degradation. The relatively low identity of these enzymes with known ones indicates their novel amino-acid sequence and possible unique and special activities.

Although, several studies have reported the ability to degrade various complex polysaccharides by *Flammeovirga* bacteria, the systematic analysis of such capability in *Flammeovirga* has not been performed previously. In this study, numerous genes encoding carbohydrate-active enzymes, and the polysaccharide degradation pathways, as well as the corresponding glycometabolism pathways were deduced. All of these enzymes belong to GHs (a total of 483) and PLs (a total of 35). Besides of agarase genes, abundant genes encoding polysaccharide-degrading enzymes were also annotated in the genome of *F. pacifica* WPAGA1, including amylase, xylosidase, cellulase, alginate lyase, pectate lyase, rhamnogalacturonan lyase, chitinase, carrageenase, and heparinase (Table 4). Accordingly, the strain WPAGA1 could secrete corresponding enzymes to degrade and utilize chitin, arabinogalactan, carrageenan, porphyran, fucoidan, cellulose, amylum, and xylan as sole carbon substrate for growing, except of heparin and pectin. Multiple enzymes perform the same function, as exemplified by the presence of 13 β-agarases in strain WPAGA1 that catalyze the hydrolysis of (1→4)-β-D-galactosidic linkages in agarose. Seemingly, these isoenzymes in the strain WPAGA1 are wasteful, but their presence may reflect multiple specificities essential for the effective hydrolysis or cleavage of complex polysaccharides. Redundant activities may indicate the need of the microorganism for biochemical diversity to achieve optimum growth under varying and adverse environment conditions in deep sea. Furthermore, almost half of the polysaccharide-degrading enzymes (27/64) possess a signal peptide and secrete into extracellular environments because almost all polysaccharides cannot pass through the cell membrane. These results showed that *F. pacifica* WPAGA1 has perfect genomic basis for metabolization of various complex polysaccharides.

Agar is the major carbohydrate in red macroalgae (*Rhodophyta*), and is composed of neutral agarose as the major component and charged agaropectin as the minor component (Usov, 1998). The main component of agar, agarose, is a heteropolysaccharide composed of equal amounts of D-galactose and 3,6-anhydro-L-galactose (AHG). Previous studies demonstrated that agarases are enzymes that catalyze agar hydrolysis, and are classified into α-agarases (EC 3.2.1.158) and β-agarases (EC3.2.1.81) depending on their cleavage actions. β-agarase and α-agarase hydrolyse the β-1,4 and α-1,3-linkages of agarose, respectively. A total of 13 agarase genes were found from the genome sequences of strain WPAGA1. It is interesting that all of them belonged to β-agarases. Previous reports have showed that most of known agarase belong to β- agarases, while α-agarases are rare (Young et al., 1978; Ohta et al., 2005). It could be concluded that β-agarase is sufficient to metabolize agar in bacteria (Figure 4).

Besides of polysaccharide-degrading enzymes, there are many other genes to assist strain WPAGA1 to degrade complex polysaccharides. Based on the genomic sequence, we found that the PPP, galactose metabolic pathway, starch and sucrose metabolism, fructose and mannose metabolism and pentose and glucoronate interconversion are involved in the further utilization of these polysaccharides. Some genes of the *F. pacifica* WPAGA1 genome encode polysaccharide deacetylase. For example, FlaGM001418 encode putative chitin deacetylase 1, which deacetylates polysaccharide. The deacetylated polysaccharide is subsequently broken down by polysaccharide-degrading enzymes. Thus, in addition to genes that directly degrade polysaccharides, *F. pacifica* WPAGA1 also harbors many other genes that encode carbohydrate related enzymes. To fully understand the polysaccharides utility mechanism, the carbohydrate related enzymes need to be further investigated in the future.

Compared with other bacterium, *F. pacifica* WPAGA1 exhibited excellent ability of complex polysaccharides degradation. Although in many previous studies, a lot of enzyme-encoding genes were found in bacteria genome, but many of these genes are silent. Interestingly, in our study, most of the annotated polysaccharides-degrading enzymes have been detected to be active. Thus, we proposed that the strain WPAGA1 could serve as a potential model for complex glycometabolism investigation. By achieving the *F. pacifica* WPAGA1 genome, we expect to gain increased understanding of the processes by which *Flammeovirga* bacterial strains colonize the deep sea, interact with other organisms in the deep-sea ecosystem and perform vital functions in the marine carbon cycle, and to gain valuable insights into the genomic basis of the species' unusual capabilities in polysaccharide degradation and carbohydrate anabolism. On the other hand, given their polysaccharide-degrading ability, the *Flammeovirga* bacteria and their enzymes can be used to efficiently biodegrade various polysaccharides to generate oligosaccharides with several bioactivities that are beneficial to the food, cosmetic and medical industries. In this context, WPAGA1 genome analysis can provide abundant novel enzymes for biodegrading various polysaccharides that may hold potential applications in the industries.

## Author notes

As technologies improve, genome sequencing has gradually become an essential step for study of a novel species. Here, we took advantage of recent technologies to examine the genome of a novel deep-sea bacterium *F. pacifica* WPAGA1. By genome sequencing, we presented the fine genome of *F. pacifica* WPAGA1, which contained many specific genomic elements and rich carbohydrate associated genes. According to gene prediction, we found exceptionally rich genes encoding carbohydrate enzymes, especially genes encoding polysaccharides-degrading enzymes, in the *F. pacifica* WPAGA1 genome. Based on these putative carbohydrate enzymes, we deduced various glycometabolic pathways, including a first proposed systemic agar-degrading metabolic pathway. These findings are particularly important because strain WPAGA1 may serve as a potential candidate for research of glycometabolism and have potential biotechnological and industrial applications.

## Author contributions

Conceptualization: BG, RZ. Formal Analysis: BG, WQ, and MJ. Funding Acquisition: RZ. Investigation: BG and LL. Methodology: BG and RZ. Project administration: RZ. Resources: BG, LL, and RZ. Supervision: RZ. Validation: BG, WQ, and LL. Visualization: BG, MJ, and RZ. Writing–Original Draft Preparation: RZ. Writing–Review & Editing: BG, MJ, and RZ.

### Conflict of interest statement

The authors declare that the research was conducted in the absence of any commercial or financial relationships that could be construed as a potential conflict of interest.

## Supplementary material

Figure S1 **Metabolism of D-galacturonate, D-xylose, and L-arabinose involved in the pentose and glucoronate interconversion pathway in ***F. pacifica*** WPAGA1**. Dotted arrows denote non-annotated corresponding enzymes.

Figure S2 **Metabolism of L-rhamnose, L-fucose, and D-mannose involved in the fructose and mannose metabolic pathways in ***F. pacifica*** WPAGA1**. Dotted arrows denote non-annotated corresponding enzymes.

Figure S3 **Metabolism of D-xylose and D-ribose involved in the PPP in ***F. pacifica*** WPAGA1**. Dotted arrows non-annotated corresponding enzymes.

Figure S4 **Metabolism of cellulose and amylose in ***F. pacifica*** WPAGA1**. Dotted arrows denote non-annotated corresponding enzymes.

Figure S5 **Metabolism of D-galactose in ***F. pacifica*** WPAGA1**. Dotted arrows denote non-annotated corresponding enzymes.

Table S1 **Genes related to polysaccharide metabolism in ***F. pacifica*** WPAGA1**.
