# Supplementary figures and images for: Genome Sequencing Reveals the Complex Polysaccharide-Degrading Ability of Novel Deep-Sea Bacterium Flammeovirga pacifica WPAGA1
Source: Front Microbiol. 2017 Apr 10;8:600. doi: 10.3389/fmicb.2017.00600 (PMC5385347; doi:10.3389/fmicb.2017.00600)

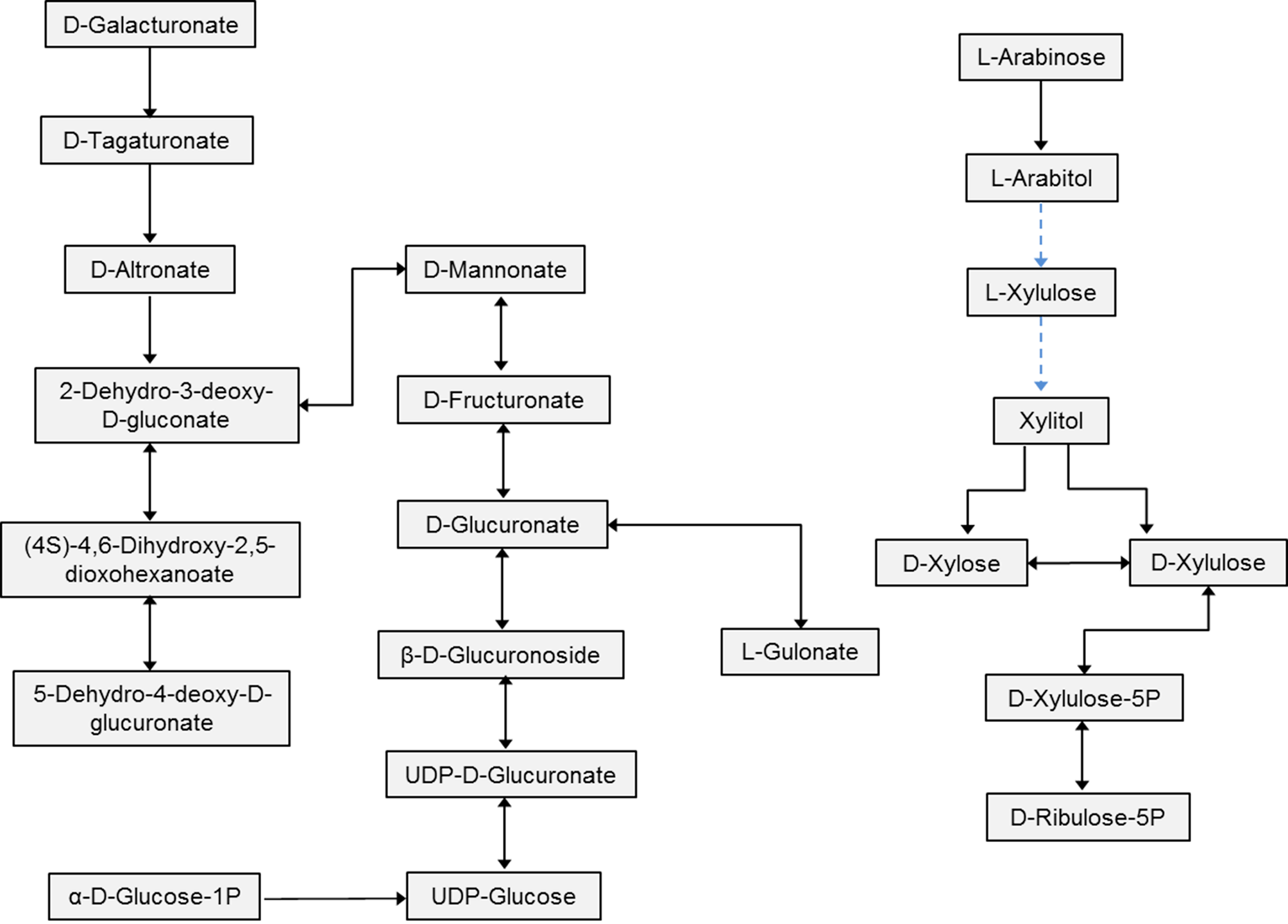

Supplement: Figure S1 — Metabolism of D-galacturonate, D-xylose, and L-arabinose involved in the pentose and glucoronate interconversion pathway in F. pacifica WPAGA1. Dotted arrows denote non-annotated corresponding enzymes. [file Image1.TIF]

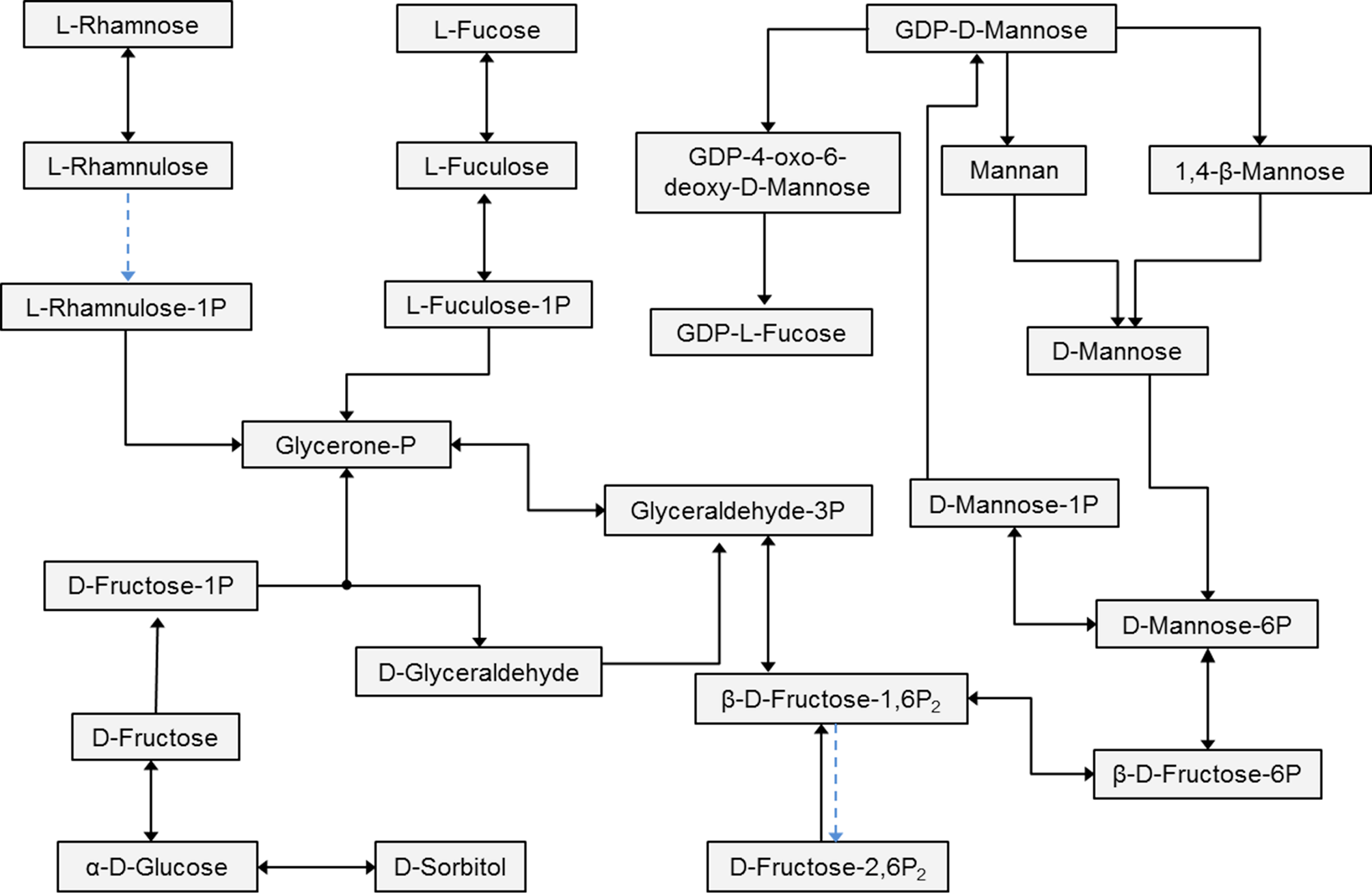

Supplement: Figure S2 — Metabolism of L-rhamnose, L-fucose, and D-mannose involved in the fructose and mannose metabolic pathways in F. pacifica WPAGA1. Dotted arrows denote non-annotated corresponding enzymes. [file Image2.TIF]

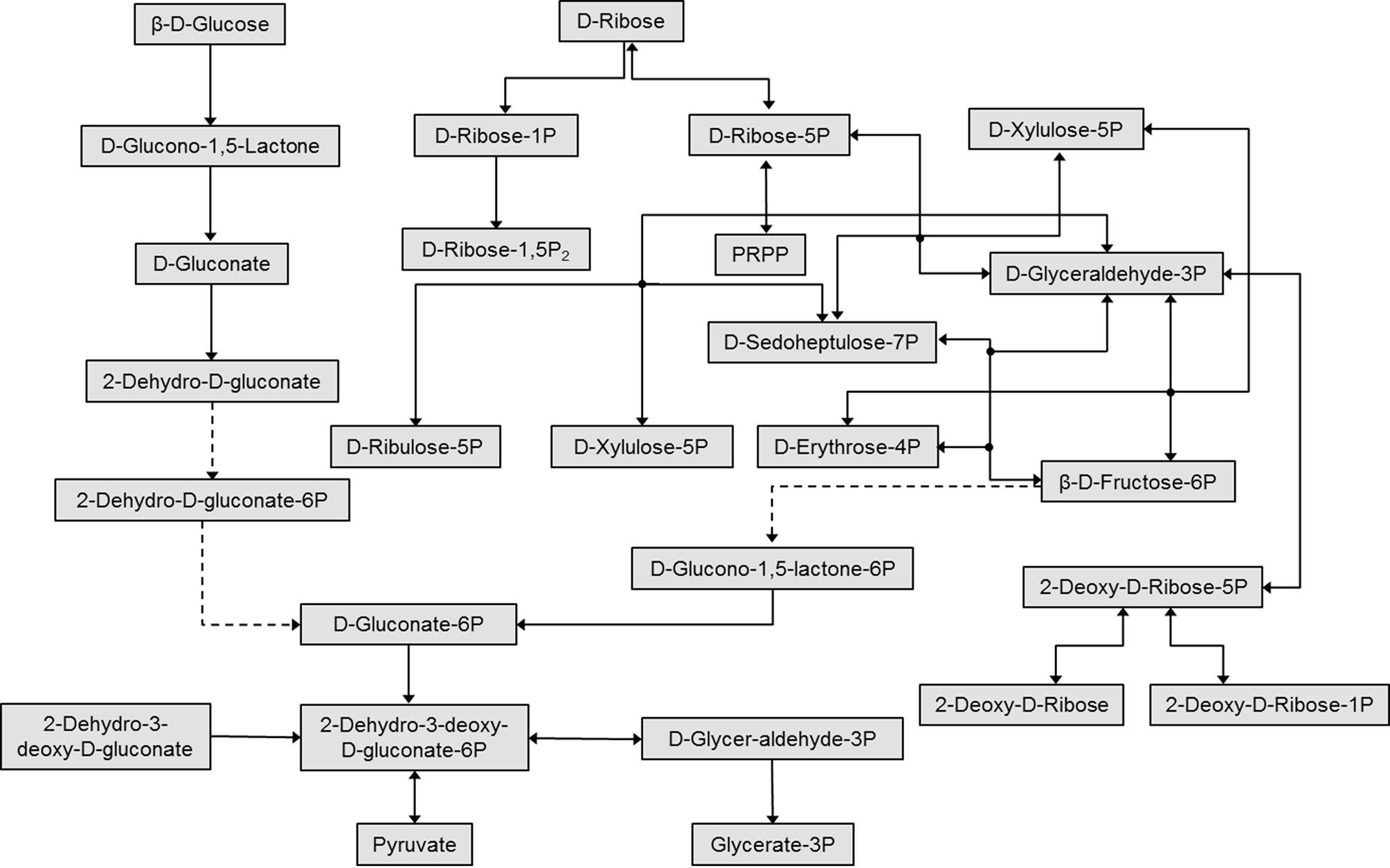

Supplement: Figure S3 — Metabolism of D-xylose and D-ribose involved in the PPP in F. pacifica WPAGA1. Dotted arrows non-annotated corresponding enzymes. [file Image3.TIF]

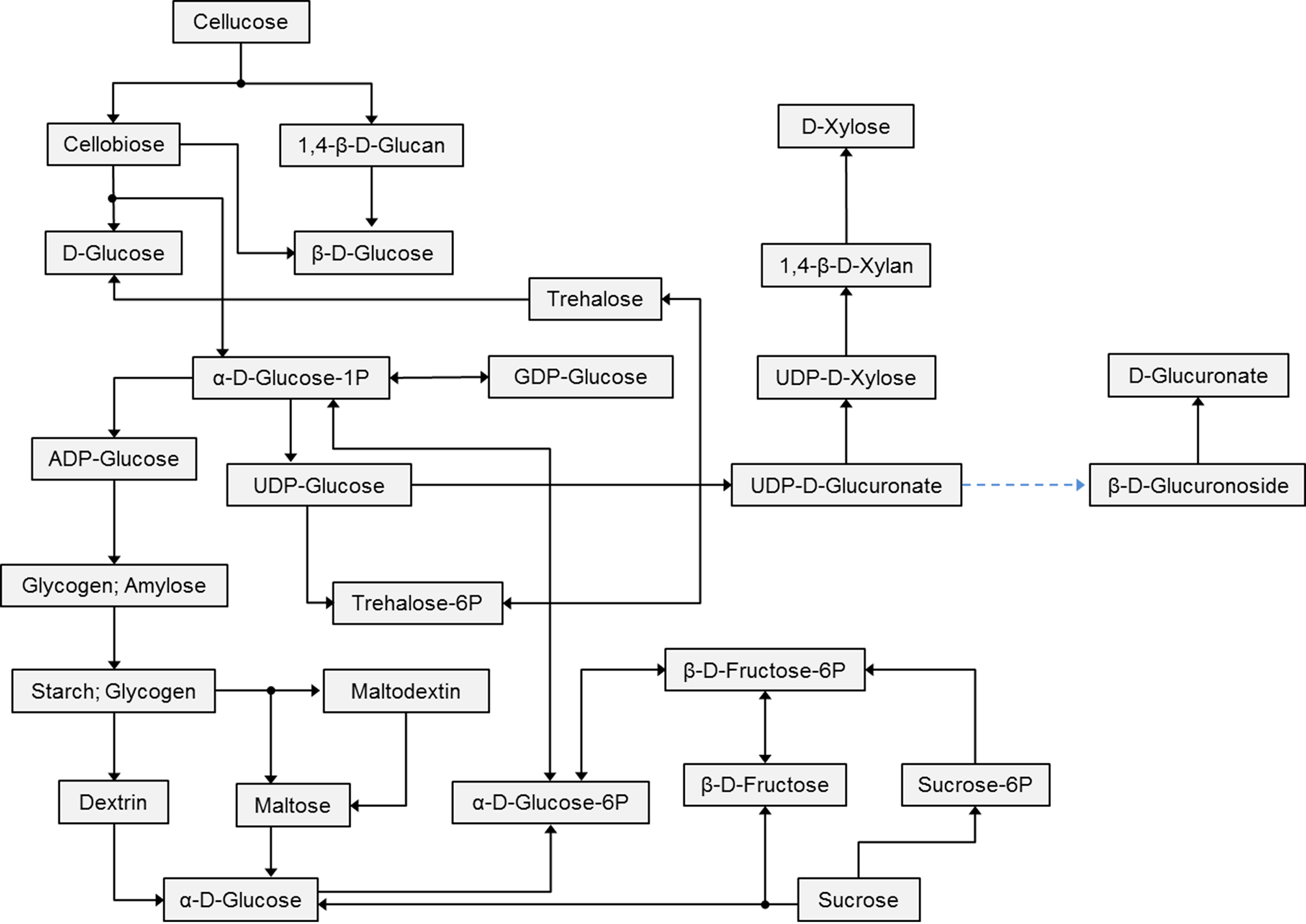

Supplement: Figure S4 — Metabolism of cellulose and amylose in F. pacifica WPAGA1. Dotted arrows denote non-annotated corresponding enzymes. [file Image4.TIF]

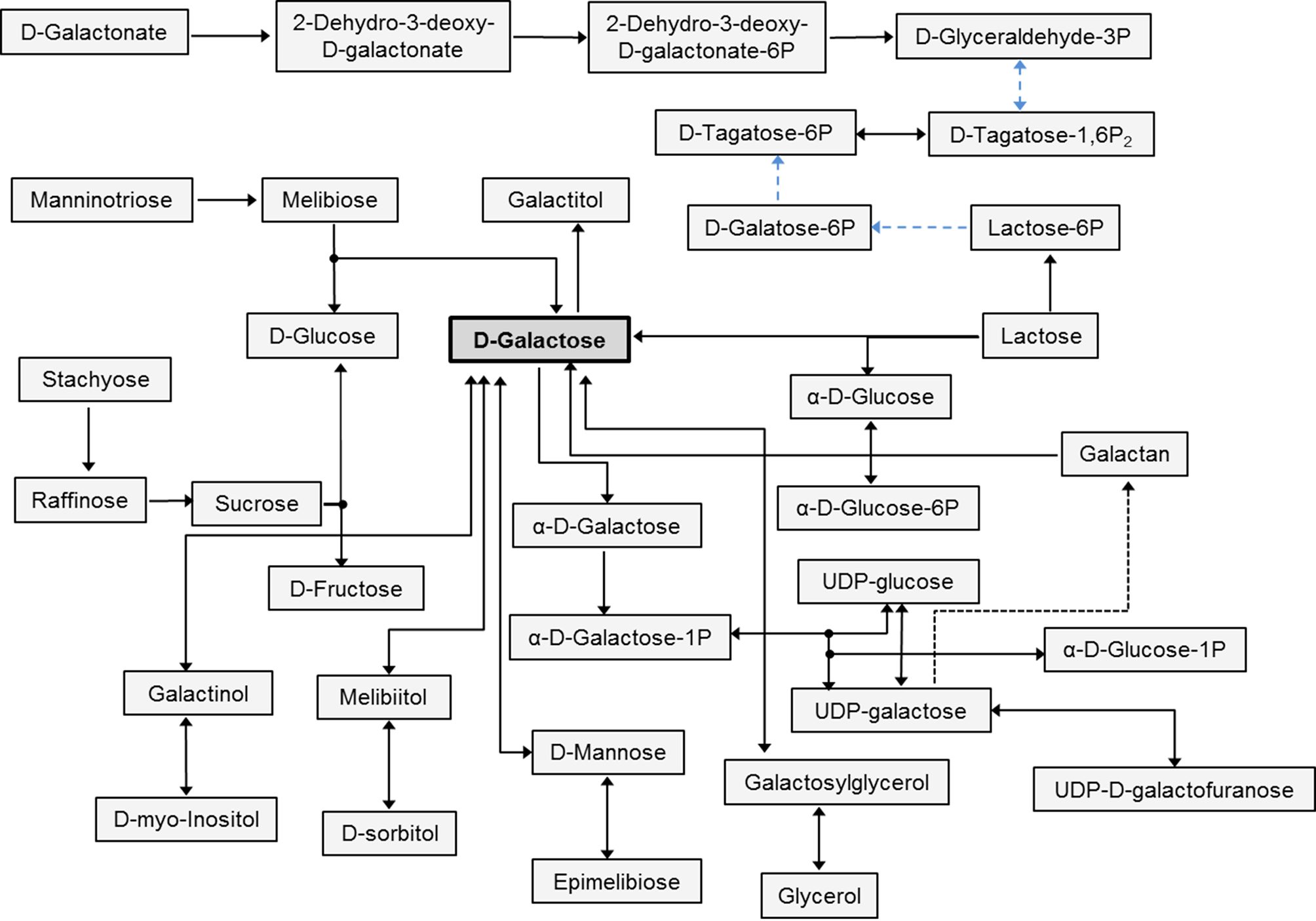

Supplement: Figure S5 — Metabolism of D-galactose in F. pacifica WPAGA1. Dotted arrows denote non-annotated corresponding enzymes. [file Image5.TIF]
