# Supplementary material for: Genome Sequencing Reveals the Complex Polysaccharide-Degrading Ability of Novel Deep-Sea Bacterium Flammeovirga pacifica WPAGA1
Source: Front Microbiol. 2017 Apr 10;8:600. doi: 10.3389/fmicb.2017.00600 (PMC5385347; doi:10.3389/fmicb.2017.00600)
Supplement: Table S1 — Genes related to polysaccharide metabolism in F. pacifica WPAGA1. [file Table1.DOCX]

**S1 Table 1** Genes related the metabolism of polysaccharide in *F. pacifica* WPAGA1.

| **Polysaccharide** | **No.** | **Related enzymes** | **Identity%** | **Family** |
| --- | --- | --- | --- | --- |
| Amylum | FlaGM000248 | α-amylase [*Draconibacterium orientale*] | 40.75 | GH13 |
|  | FlaGM000249 | α-amylase [*Bacteroides dorei*] | 40.66 | GH13 |
|  | FlaGM001439 | α-amylase [*Emticicia oligotrophica* DSM 17448] | 53.03 | GH13 |
|  | FlaGM001694 | α-amylase [*Chloroherpeton thalassium* ATCC 35110] | 39.79 | GH13 |
|  | FlaGM001879 | α-amylase [*Paenibacillus riograndensis* SBR5] | 39.54 | GH13 |
|  | FlaGM002138 | α-amylase [*Cyanothece* sp. PCC 7822] | 41.71 | GH13 |
|  | FlaGM002782 | α-amylase [*Flammeovirga pacifica*] | 100 | GH13 |
|  | FlaGM002923 | β-amylase [*Geodermatophilus obscurus* DSM 43160] | 41.23 | GH14 |
| Xylan | FlaGM001213 | Xylanase [*Cytophaga hutchinsonii* ATCC 33406] | 27.45 | GH8 |
|  | FlaGM001714 | Xylan 1,4-β-xylosidase [*Paenibacillus mucilaginosus* K02] | 27.97 | GH39 |
|  | FlaGM003088 | Endo-1,3-β-xylanase [*Psychroflexus torquis* ATCC 700755] | 41.3 | GH26 |
|  | FlaGM003092 | Endo-1,3-β-xylanase [*Cellulophaga algicola* DSM 14237] | 57.39 | GH26 |
|  | FlaGM003314 | Xylan 1,4-β-xylosidase [*Paenibacillus mucilaginosus* 3016] | 31.13 | GH39 |
|  | FlaGM003805 | Xylan 1,4-β-xylosidase [*Staphylococcus aureus* subsp. aureus VC40] | 27.82 | GH39 |
|  | FlaGM004512 | Endo-1,3-β-xylanase [*Cellulophaga algicola* DSM 14237] | 56.23 | GH26 |
|  | FlaGM004515 | Xylan 1,4-β-xylosidase [*Paludibacter propionicigenes* WB4] | 76.03 | GH43 |
|  | FlaGM004517 | Xylanase [*Flavobacterium johnsoniae* UW101] | 41.63 | GH10 |
|  | FlaGM004522 | Endo-1,4-β-xylanase [*Pseudopedobacter saltans* DSM 12145] | 52.97 | GH10 |
| Cellulose | FlaGM002317 | Cellulose 1,4-β-cellobiosidase [*Dyadobacter fermentans* DSM 18053] | 30.26 | GH9 |
|  | FlaGM002741 | Cellulase [uncultured bacterium BLR10] | 33.33 | GH9 |
| Alginate | FlaGM002034 | Alginate lyase [*Niastella koreensis* GR20-10] | 32.14 | PL7 |
|  | FlaGM002039 | Alginate lyase [*Flammeovirga* sp. MY04] | 26.07 | PL7 |
|  | FlaGM002592 | Alginate lyase [*Zobellia galactanivorans*] | 66.76 | PL7 |
|  | FlaGM004227 | Alginate lyase [*Flammeovirga* sp. MY04] | 76.54 | PL7 |
| Agar | FlaGM001950 | β-agarase [*Flammeovirga* sp. OC4] | 60.47 | GH86 |
|  | FlaGM001957 | β-agarase [*Flammeovirga* sp. OC4] | 68.73 | GH86 |
|  | FlaGM001981 | β-agarase [*Cellulophaga algicola* DSM 14237] | 32.11 | GH86 |
|  | FlaGM002050 | β-agarase [*Flammeovirga* sp. OC4] | 73.07 | GH86 |
|  | FlaGM002515 | Agarase [*Gilvimarinus polysaccharolyticus*] | 46.89 | GH86 |
|  | FlaGM002593 | β-agarase [*Microscilla* sp. PRE1] | 37.88 | GH86 |
|  | FlaGM002660 | β-agarase [*Coraliomargarita akajimensis* DSM 45221] | 40.62 | GH50 |
|  | FlaGM004007 | β-agarase [*Microscilla* sp. PRE1] | 44.76 | GH86 |
|  | FlaGM004591 | Agarase [*Flammeovirga yaeyamensis*] | 87.14 | GH16 |
|  | FlaGM004871 | β-agarase [*Microscilla* sp. PRE1] | 37.46 | GH86 |
|  | FlaGM004902 | Agarase B8 [*Flammeovirga yaeyamensis*] | 85.16 | GH86 |
|  | FlaGM004974 | β-agarase [*Microscilla* sp. PRE1] | 38.46 | GH86 |
|  | FlaGM004975 | β-agarase [*Microscilla* sp. PRE1] | 48.18 | GH86 |
|  | FlaGM004900 | Glycoside hydrolase [*Flammeovirga yaeyamensis*] | 91.77 | GH117 |
|  | FlaGM004986 | AHG dehydrogenase [*Vibrio* sp. EJY3] | 73 | Unidentified |
|  | FlaGM004985 | AHGA cycloisomerase [*Vibrio* sp. EJY3] | 64 | Unidentified |
|  | FlaGM004649 | AHGA cycloisomerase [*Vibrio* sp. EJY3] | 44 | Unidentified |
| Pectate | FlaGM000253 | Pectate lyase [*Cytophaga hutchinsonii* ATCC 33406] | 23.64 | PL1 |
|  | FlaGM004148 | Pectate lyase [*Cytophaga hutchinsonii* ATCC 33406] | 23.67 | PL1 |
|  | FlaGM004069 | Pectin lyase [*Acholeplasma oculi*] | 34.93 | PL1 |
|  | FlaGM004071 | Pectin lyase [*Acholeplasma oculi*] | 32.02 | PL1 |
| Rhamnogalacturonan | FlaGM002940 | Rhamnogalacturonan lyase [*Cytophaga hutchinsonii* ATCC 33406] | 26.98 | PL11 |
| Chitin | FlaGM001246 | Chitinase I [*Pantoea dispersa*] | 40.74 | GH18 |
|  | FlaGM001418 | Chitin deacetylase 1 [*Marinobacter* sp. BSs20148] | 32.16 | CE4 |
|  | FlaGM001424 | Chitinase [*Peptoclostridium difficile*] | 34.31 | GH18 |
|  | FlaGM001461 | Chitinase [*Peptoclostridium difficile* R20291] | 33.33 | GH18 |
|  | FlaGM001949 | Endo-chitinase [*Microbulbifer hydrolyticus*] | 34.72 | GH18 |
|  | FlaGM002003 | Chitinase [*Clostridium sordellii*] | 30.72 | GH18 |
|  | FlaGM002006 | Chitinase [*Clostridium sordellii*] | 30.12 | GH18 |
|  | FlaGM002033 | Chitinase [*Enterococcus mundtii* QU 25] | 34.36 | GH18 |
|  | FlaGM003562 | Chitinase [*Shewanella violacea* DSS12] | 38.62 | GH18 |
|  | FlaGM003618 | Chitin deacetylase [*Flexibacter litoralis* DSM 6794] | 53.02 | CE4 |
|  | FlaGM003813 | Chitinase [*Ophiocordyceps sinensis*] | 25.17 | GH18 |
| Heparin | FlaGM002044 | heparinase II/III-like protein [*Polaribacter* sp. MED152] | 48.63 | PL17 |
| Carrageen | FlaGM003973 | κ-carrageenase [*Rhodopirellula baltica* SH 1] | 40.96 | GH16 |
|  | FlaGM003999 | λ-carrageenase [*Pseudoalteromonas* sp. CL19] | 46.41 | PL11 |
|  | FlaGM004000 | ι-carrageenase [*Zobellia galactanivorans*] | 45.77 | GH82 |
|  | FlaGM004113 | ι-carrageenase [*Microbulbifer thermotolerans*] | 28.12 | GH82 |
|  | FlaGM004431 | ι-carrageenase [*Zobellia galactanivorans*] | 42.82 | GH82 |
|  | FlaGM004436 | λ-carrageenase [*Pseudoalteromonas* sp. CL19] | 45.71 | PL11 |
|  | FlaGM004624 | ι-carrageenase [*Zobellia galactanivorans*] | 42.71 | GH82 |
|  | FlaGM004753 | ι-carrageenase [*Zobellia galactanivorans*] | 43.59 | GH82 |
| Fucoidin | FlaGM001676 | α-L-fucosidase [*Draconibacterium orientale*] | 70.13 | GH29 |
|  | FlaGM001951 | α-L-fucosidase [*Cellulophaga lytica*] | 64.65 | GH29 |
|  | FlaGM001958 | α-L-fucosidase [*Cellulophaga lytica*] | 61.93 | GH29 |
|  | FlaGM001980 | α-L-fucosidase [*Cellulophaga lytica*] | 57.82 | GH29 |
|  | FlaGM002148 | α-L-fucosidase [*Maribacter* sp. HTCC2170] | 57.72 | GH29 |
|  | FlaGM004202 | α-L-fucosidase [*Akkermansia muciniphila* ATCC BAA-835] | 36.16 | GH29 |
|  | FlaGM004254 | α-L-fucosidase [*Formosa agariphila* KMM 3901] | 66.4 | GH29 |
|  | FlaGM004260 | α-L-fucosidase [*Cellulophaga lytica*] | 62.26 | GH29 |
|  | FlaGM004261 | α-L-fucosidase [*Cellulophaga lytica*] | 38.18 | GH29 |
|  | FlaGM004297 | α-L-fucosidase [*Pedobacter heparinus* DSM 2366] | 43.54 | GH29 |
|  | FlaGM004768 | α-L-fucosidase [*Formosa agariphila* KMM 3901] | 66.72 | GH29 |
|  | FlaGM004770 | α-L-fucosidase [*Bifidobacterium bifidum* BGN4] | 28.87 | GH95 |
|  | FlaGM004881 | α-L-fucosidase [*Sphingobacterium* sp. 21] | 58.54 | GH95 |
|  | FlaGM004882 | α-L- fucosidase [*Coraliomargarita akajimensis* DSM 45221] | 36.05 | GH29 |
|  | FlaGM004910 | α-L-fucosidase [*Algibacter* sp. HZ22] | 69.07 | GH29 |
|  | FlaGM004929 | α-L-fucosidase [*Coraliomargarita akajimensis* DSM 45221] | 64.6 | GH29 |
|  | FlaGM005001 | α-L-fucosidase [*Sphingobacterium* sp. 21] | 53.81 | GH29 |
| The other  polysaccharides | FlaGM000635 | Endo-Β-1,4-Galactanase [*Streptococcus gallolyticus* UCN34] | 34.87 | GH53 |
|  | FlaGM000887 | Polysaccharide deacetylase [Candidatus *Koribacter versatilis* Ellin345] | 25.1 | CE4 |
|  | FlaGM000981 | Polysaccharide deacetylase [Candidatus *Koribacter versatilis* Ellin345] | 25.73 | CE4 |
|  | FlaGM001153 | Polysaccharide deacetylase [*Janthinobacterium agaricidamnosum*] | 27.71 | CE4 |
|  | FlaGM001354 | polysaccharide deacetylase [*Xylella fastidiosa* MUL0034] | 28.85 | CE4 |
|  | FlaGM002015 | β-porphyranase D [*Zobellia galactanivorans*] | 28.4 | GH16 |
|  | FlaGM002946 | pectinesterase [*Clavibacter michiganensis* subsp. nebraskensis] | 25.82 | CE8 |
|  | FlaGM003843 | Pectinesterase [*Pseudopedobacter* *saltans* DSM 12145] | 30.95 | CE8 |
|  | FlaGM003960 | Pectinesterase [*Pseudopedobacter saltans* DSM 12145] | 31.63 | CE8 |
|  | FlaGM004577 | β-1,4-mannanase [*Flammeovirga yaeyamensis*] | 78.86 | GH5 |
|  | FlaGM004586 | β-mannanase [*Flammeovirga yaeyamensis*] | 84.66 | GH26 |
|  | FlaGM004582 | Endo-1,4-β-mannosidase [*Flammeovirga yaeyamensis*] | 80.14 | GH5 |
|  | FlaGM004710 | Endoglucanase-related protein [*Cytophaga hutchinsonii* ATCC 33406] | 33.64 | CBM51;CBM6 |
|  | FlaGM004735 | Pectinesterase [*Rhodothermus marinus* SG0.5JP17-172] | 30.05 | CE8 |
|  | FlaGM004949 | Arabinogalactan endo-1,4-β-galactosidase [*Belliella baltica* DSM 15883] | 49.35 | GH53 |
